# Promoting collateral formation in type 2 diabetes mellitus using ultra-small nanodots with autophagy activation and ROS scavenging

**DOI:** 10.1186/s12951-024-02357-z

**Published:** 2024-03-01

**Authors:** Yixuan Wang, Feifei Li, Linshuang Mao, Yu Liu, Shuai Chen, Jingmeng Liu, Ke Huang, Qiujing Chen, Jianrong Wu, Lin Lu, Yuanyi Zheng, Weifeng Shen, Tao Ying, Yang Dai, Ying Shen

**Affiliations:** 1 Department of Cardiovascular Medicine, Rui Jin Hospital, Shanghai Jiao Tong University School of Medicine, Shanghai, 200025 China; 2 Shanghai Clinical Research Center for Interventional Medicine, Shanghai, 200025 China; 3 Beijing Advanced Innovation Center for Big Data-Based Precision Medicine, School of Medicine and Engineering, Beihang University, Beijing, 100191 China; 4 Department of Ultrasound in Medicine, Shanghai Sixth People’s Hospital, Shanghai Jiao Tong University School of Medicine, Shanghai, 200233 China

**Keywords:** Molybdenum disulfide nanodots, Type 2 diabetes mellitus, Collateral formation, Reactive oxygen species, Autophagy

## Abstract

**Background:**

Impaired collateral formation is a major factor contributing to poor prognosis in type 2 diabetes mellitus (T2DM) patients with atherosclerotic cardiovascular disease. However, the current pharmacological treatments for improving collateral formation remain unsatisfactory. The induction of endothelial autophagy and the elimination of reactive oxygen species (ROS) represent potential therapeutic targets for enhancing endothelial angiogenesis and facilitating collateral formation. This study investigates the potential of molybdenum disulfide nanodots (MoS_2_ NDs) for enhancing collateral formation and improving prognosis.

**Results:**

Our study shows that MoS_2_ NDs significantly enhance collateral formation in ischemic tissues of diabetic mice, improving effective blood resupply. Additionally, MoS_2_ NDs boost the proliferation, migration, and tube formation of endothelial cells under high glucose/hypoxia conditions in vitro. Mechanistically, the beneficial effects of MoS_2_ NDs on collateral formation not only depend on their known scavenging properties of ROS (H_2_O_2_, •O_2_^-^, and •OH) but also primarily involve a molecular pathway, cAMP/PKA-NR4A2, which promotes autophagy and contributes to mitigating damage in diabetic endothelial cells.

**Conclusions:**

Overall, this study investigated the specific mechanism by which MoS_2_ NDs mediated autophagy activation and highlighted the synergy between autophagy activation and antioxidation, thus suggesting that an economic and biocompatible nano-agent with dual therapeutic functions is highly preferable for promoting collateral formation in a diabetic context, thus, highlighting their therapeutic potential.

**Graphical Abstract:**

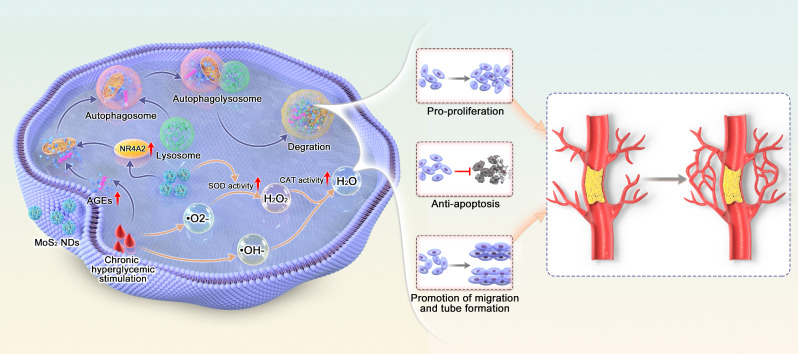

**Supplementary Information:**

The online version contains supplementary material available at 10.1186/s12951-024-02357-z.

## Background

Type 2 diabetes mellitus (T2DM) accelerates the progression of atherosclerotic cardiovascular diseases (ASCVDs). ASCVDs, including coronary artery disease and peripheral artery disease, lead to cardiac dysfunction and lower limb amputation due to occlusions of arteries [1, 2]. Studies suggest that when an artery is occluded, collaterals – the arteriolar networks connecting arteries in tissues such as the heart and skeletal muscle – serve as a conduit, which can partially ensure blood supply, prevent or alleviate tissue ischemia, and reduce complications [3–5]. However, collaterals in patients with T2DM are also weaker than those in non-diabetic patients due to more severe and diffused artery atherosclerosis and impaired endothelial cell function [6, 7].

Impaired collateral formation leads to poor prognosis of T2DM patients with ASCVDs [8]. Attempts to improve collateral formation in diabetes have failed to achieve the expected results [9, 10]. Pro-angiogenic factors, including vascular endothelial growth factor (VEGF), fibroblast growth factor (FGF), and platelet-derived growth factor (PDGF), are recognized as highly effective substances in promoting collateral formation [9, 11]. However, under diabetic conditions, there is an impairment of angiogenic signaling pathways, accompanied by the overactivation of the ubiquitin-proteasome pathway and the downregulation of their respective receptors [12, 13]. These changes result in the suboptimal efficacy of pro-angiogenic factor-based therapies. Furthermore, cell-based therapies [14] aiming to promote collateral formation also encounter obstacles. Factors such as hyperglycemia, ischemia, and inflammation within the diabetic environment can negatively impact the survival and functionality of transplanted cells, which leads to significant impairments in the effectiveness of stem cell-mediated repair [15]. Therefore, the exploration and development of new drugs and approaches to address the negative impact of the diabetic environment and endothelial dysfunction to enhance collateral formation are urgently needed.

High glucose and advanced glycation end products (AGEs) accumulation lead to an overload of reactive oxygen species (ROS) [16], and inhibition of cellular autophagy [17], representing the two primary pathological factors underlying endothelial cell dysfunction in diabetes. ROS overload leads to cellular inflammatory response [18], DNA damage [19], and lipid peroxidation in the cell membrane [20], ultimately triggering endothelial cell apoptosis. Autophagy inhibition leads to the failure to clear damaged substances [21], intensifying intracellular oxidative stress and inflammation [22, 23], impacting cellular energy metabolism [24], and disrupting the normal physiological functions of endothelial cells, including their survival, proliferation, migration, and sprouting capabilities [25]. Therefore, reducing ROS levels and activating autophagy both play roles in maintaining the homeostasis of endothelial cells, thereby protecting endothelial cell function and improving collateral formation [26, 27].

**Fig. 1:**
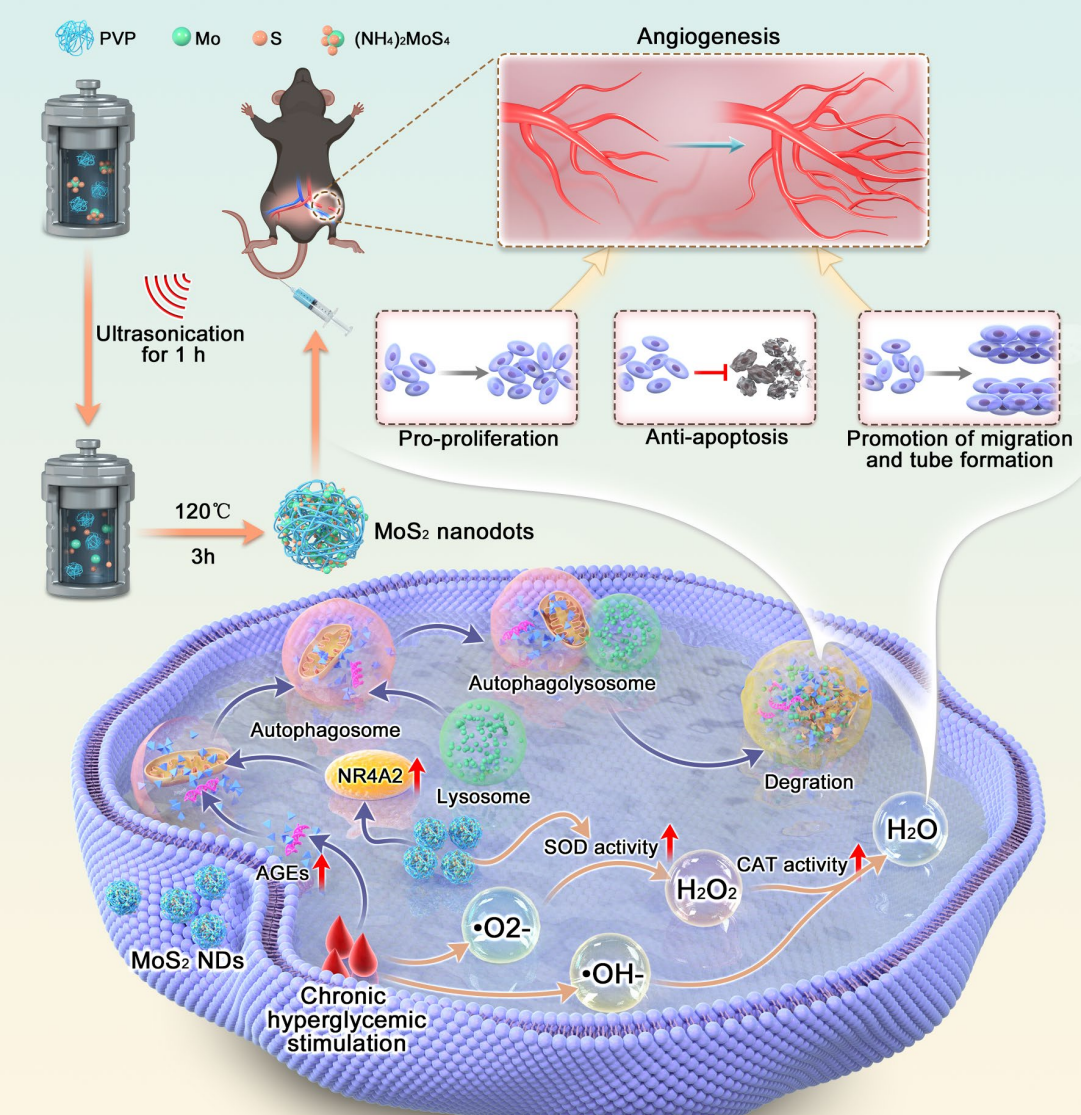
Schematic showing MoS_2_ NDs mediated diabetic neovascularization. The illustration shows that MoS_2_ NDs promote angiogenesis by restoring the blood flow of the lower limb in diabetic mice after HLI, as well as facilitating endothelial cell migration, tube formation, and proliferation but inhibiting apoptosis. The antioxidant enzyme activity and autophagy activation properties of MoS_2_ NDs have been proposed as potential mechanisms for improving impaired angiogenesis in diabetes

Inorganic nanoparticles have been developed and utilized in therapy for various diseases owing to their outstanding advantages, including high stability, long blood half-life, and multiple enzyme-like activities compared with traditional medication [28–30]. As a two-dimensional transition metal dichalcogenide, molybdenum disulfide (MoS_2_) nanosheets have found widespread applications in anti-inflammatory and antibacterial therapies [31]. To enhance the biocompatibility of MoS_2_ materials, sub-10-nanometer ultra-small MoS_2_ nanodots (MoS_2_ NDs) have been developed [32, 33]. These nanodots not only retain the distinctive ROS scavenging properties of MoS_2_ [34] but also demonstrate higher clearance rates and less retention in vivo [35, 36]. It is noteworthy that recent studies have revealed the significant enhancement of cellular autophagy activity by MoS_2_ nanomaterials [37, 38]. Considering the crucial roles of ROS overload and autophagy inhibition in diabetic vascular diseases, we speculate that MoS_2_ nanodots with dual functions in ROS scavenging and autophagy activation may serve as effective means to intervene in diabetic collateral formation [39, 40]. Therefore, we prepared MoS_2_ NDs using a simple solvothermal method and assessed their characteristics, such as body clearance rate, biocompatibility, and catalytic properties. To comprehensively assess the effect of MoS_2_ NDs on collateral formation in diabetes, we established diabetic mouse hindlimb ischemia (HLI) models and high glucose/hypoxia (HG/Hypo) endothelial cell models treated with MoS_2_ NDs. Various indicators, including angiogenic capacity after ischemia, endothelial cell function, and autophagic activity, were examined. Additionally, by using omics analysis and molecular biology techniques, we further revealed a novel molecular mechanism by which MoS_2_ NDs activate endothelial autophagy, involving the upregulation of the cAMP/PKA-NR4A2 pathway. Overall, this study is expected to provide potential therapeutic approaches for treating impaired collateral formation in diabetes (Fig. 1).

## Methods

### Cell culture

Primary human umbilical vein endothelial cells (HUVECs) were obtained from ScienCell (Cas# 8000, ScienCell, USA) and cultured at 37 °C in endothelial culture medium containing 5% fetal bovine serum (FBS) (Cas# 0025, ScienCell), 1% endothelial cell growth supplement (ECGS) (Cas# 1052, ScienCell), and 1% penicillin/ Streptomycin solution (Cas# 0503, ScienCell). The second to eighth passages of HUVECs were used for subsequent experiments.

### Animal feeding

C57BL/6 mice (males, 7–8 weeks) were obtained from the Shanghai JASJ Laboratory Animal Co. All animal experiments were performed in compliance with the National Institutes of Health Guide for the Care and Use of Laboratory Animals (NIH Publication No. 85 − 23, revised 1996) and were approved by the Animal Resources Committee of Shanghai Jiao Tong University. All mice were housed in a pathogen-free setting at the Animal Experimentation Center of Ruijin Hospital, Shanghai Jiao Tong University School of Medicine. The animals were given free access to food and water during the 12 h light/dark cycle.

### MoS_2_ NDs preparation

MoS_2_ NDs were synthesized according to a previously described method [35]. Briefly, 150 mg of polyvinyl pyrrolidone (PVP) (MW: 10,000, Cas# 9003-39-8, Sigma-Aldrich, USA) and 25 mg of ammonium tetrathiomolybdate [(NH_4_)_2_MoS_4_] (Cas# 15060-55-6, Sigma-Aldrich) were mixed in 80 mL of methanol. Next, 0.80 mL of hydrazine hydrate (Cas# 10217-52-4, Sigma-Aldrich) was added dropwise. The mixture was ultrasonicated for 1 h, then placed in a stainless-steel autoclave lined with Teflon, and was allowed to react at 120 °C for 3 h. Subsequently, the dispersion was filtered, and methanol was removed using a rotary evaporator. The final dialyzable dispersion was obtained by dialyzing the result against deionized water six times for 72 h. The morphology of MoS_2_ NDs was assessed by Transmission electron microscopy (TEM) (JEM-2100 F, Japan Electronics Co., Ltd.). D8 ADVANCE X-ray Diffraction Instrument (Bruker, USA) was used to measure the X-ray Diffraction (XRD) pattern of MoS_2_ NDs. An ESCAlab250 electron spectrometer (Thermo Fisher Scientific, USA) was used to obtain X-ray photoelectron spectroscopy (XPS). The hydrodynamic sizes of the nanoparticles were characterized using a Nano ZS 90 Malvern Zetasizer Nano Series (Malvern, UK). An Evolution 350 (Thermo Fisher Scientific, USA) was used to obtain the UV–vis absorption spectra of the MoS_2_ NDs. For fluorescein isothiocyanate (FITC) labeling, MoS_2_ NDs aqueous solution (10 mL) was mixed with an ethanolic solution of FITC-PEG-SH (0.1 mg/mL). The mixture was stirred for 12 h in the dark, followed by dialysis against deionized water for 3 days to obtain the FITC-labeled MoS_2_ NDs.

### Hemolysis assay

Blood was mixed with PBS (10 mM) and then centrifuged at 3000 rpm for 5 min. This process is repeated 3–4 times to get erythrocytes. The precipitated erythrocytes were re-dispersed in PBS and then added to of pure water (positive control), PBS (negative control), and different concentrations of MoS_2_ NDs. After that, the mixtures were cultured at 37 °C for 8 h. Subsequently, the absorbance of supernatants at 540 nm was measured by UV − vis spectra. The hemolysis rate (HR%) was calculated according to the following formula: HR% = (A _MoS2_ -A_PBS_)/(A_water_-A_PBS_) × 100%. Where A _MoS2_, A_water_, and A_PBS_ are the absorbance of the sample, the positive control, and the negative control, respectively.

### Intracellular MoS_2_ NDs detection

The intracellular MoS_2_ NDs content was detected by inductively coupled plasma optical emission spectroscopy (ICP-OES) (725-ES, Agilent). The stability of MoS_2_ NDs within cells was confirmed by bio-TEM. Briefly, MoS_2_ NDs were incubated with endothelial cells. The cells were fixed overnight in a 2.5% glutaraldehyde solution, underwent gradient dehydration, and were freeze-dried. Finally, they were placed under a transmission electron microscope for observation.

### Evaluation of the ability of MoS_2_ NDs to scavenge •OH, •O_2_^-^ and H_2_O_2_

The OH-scavenging ability of the MoS_2_ NDs was investigated using electron spin resonance (ESR) spectroscopy. Typically, OH was produced using Fenton-like reagents of 2 mM FeSO_4_ and 5 mM H_2_O_2_ for 5 min. Subsequently, different concentrations of MoS_2_ NDs and 5,5-dimethyl-1-pyrroline N-oxide (DMPO) (5 µL, 98%, Cas# 3367-61-1, Sigma-Aldrich) were added to the mixture. Thereafter, the ability of the samples to scavenge OH was determined *via* ESR spectroscopy (EMX1598, Bruker, Germany). Additionally, the •OH-scavenging ability, superoxide dismutase (SOD)-like activity, and catalase (CAT)-like activities of MoS_2_ NDs were detected using the hydroxyl radical antioxidant capacity assay kit (Cat# BC1320, Solarbio), the SOD assay kit (Cat# 9054-89-1, Boxbio, China), and the H_2_O_2_ assay kit (Cat# KTB1041, Abbkine).

### In vivo biodistribution and metabolism study

Tail vein injections of the MoS_2_ NDs were performed 0.5, 2, 6, 12, and 24 h after injections. The hearts, livers, spleens, lungs, and kidneys were removed 0.5, 2, 6, 12, and 24 h after injection. A lysis buffer of 0.5 mL was applied after the trituration of all collected tissues, and the Mo level was determined using ICP-OES. To evaluate the in vivo metabolism of the MoS_2_ NDs, mouse urine and feces were collected at different times and analyzed using ICP-OES. The distribution and metabolism in different organs, feces, and urine were expressed as percentages of the tissue-injected dose per gram (%ID/g). To examine the shape of MoS_2_ NDs in urine and feces, we collected them from 24 to 48 h after the injection of MoS_2_ NDs. urine samples from mice were centrifuged at 12,000 rpm for 10 min. The pellet was dissolved in water, and a droplet was placed on a copper grid to observe the morphology of MoS_2_ NDs by TEM. Fecal samples were collected and made into a suspension. The suspension was centrifuged first at 1000 rpm for 10 min to obtain a supernatant. The supernatant was further centrifuged at 12,000 rpm for 10 min. The resulting pellet was dissolved in water. A droplet of the suspension was placed on a copper grid for visualization.

### In vitro and in vivo biosafety and biocompatibility study

The biosafety of the MoS_2_ NDs in vitro was determined by a cell counting kit-8 (CCK-8) (Cat# 521,942, Biosharp, China). Firstly, 96-well plates were used to seed the cells (5 × 10^3^ cells/well) with varied concentrations of MoS_2_ NDs for 12 h and washed three times with PBS. Subsequently, CCK-8 reagent (10 µL) diluted in the culture medium (90 µL) was added to the wells and was incubated at 37 °C for 2 h. Lastly, a microplate analyzer (DNM-9602 A, China) was used to measure the absorbance of the cells at 450 nm. For in vivo assessment, mice were intravenously injected with PBS and MoS_2_ NDs (100 µL, 2 mg/kg) every other day. On day 7, eyeball blood was collected into an anticoagulant tube containing ethylenediaminetetraacetic acid (EDTA) (Cas# 60004, Sigma-Aldrich). After 30 min, the blood was centrifuged at 3000 × *g* for 10 min. Finally, the hearts, livers, spleens, lungs, and kidneys were collected and analyzed by H&E staining. The levels of IFN-γ, IL-1β, IL-6, and TNF-γ in the mouse serum were measured using ELISA kits (IFN-γ, Cat# SEKM-0031; IL-1β, Cat# SEKM-0002; IL-6, Cat# SEKM-0007; TNF-γ, Cat# SEKM-0034, Solarbio).

### Cellular uptake assay

HUVECs were exposed to 50 µg/mL FITC-labeled MoS_2_ NDs for 1, 2, 4, 8, 12, and 24 h. 4% paraformaldehyde (PFA) (Cat# P0099, Beyotime, China) fixative was applied next, with cells stained with 4′,6-diamidino-2-phenylindole (DAPI) (Cat# C1006, Beyotime). The fluorescence microscope (BX51, Olympus, Japan) was used for visualization. Finally, flow cytometry (CytoFLEX S, Beckerman Coulter, USA) was used to quantify the FITC intensity within the cells.

### Construction of high-fat diet (HFD)/streptozotocin (STZ)-induced type 2 diabetes mouse model

C57BL/6 mice were fed with HFD for four weeks (20% kilocalorie protein, 20% kilocalorie carbohydrate, and 60% kilocalorie fat) followed by intraperitoneal injections of 60 mg/kg STZ (Cas# 18883-66-4, Sigma-Aldrich) for 5 days. Subsequently, the mice with blood glucose levels ≥ 15.7 mM were considered diabetic and were used to establish the HLI model.

### Femoral artery ligation

HFD/STZ-induced diabetic mice were anesthetized with continuous inhalation of 2.5% isoflurane gas. The left superficial femoral artery was ligated proximal to the deep femoral artery and was ligated at the branch to the tibial artery, clipping the ligature short and leaving the nerve and vein intact. Subsequently, the blood flow during functional recovery was detected by the laser Doppler imaging instruments (MoorLDI2, Moor, UK) on days 0, 3, 7, 14, and 21. The recovery rate was measured as the blood flow on the affected and unaffected sides [41]. The limb function score was used to assess ischemic injury and mobility impairment semi-quantitatively. It was calculated as follows: ischemic damage: contralateral hindlimb = 0; mild discoloration = 1; moderate discoloration = 2; severe discoloration or partial tissue loss = 3; and any amputation = 4 [42]. Mice were injected with MoS_2_ NDs (2 mg/kg) and PBS through the tail vein every other day from 3 days preoperatively to 21 days postoperatively. Cy5.5-MoS_2_ NDs were synthesized to assess the biodistribution of MoS_2_ NDs in ischemic muscles in the hindlimb ischemia model 3 days preoperatively by the in vivo fluorescence imaging system (CleVue Basic System, Vieworks, Korea). The biodistribution of MoS_2_ NDs on the ischemic site and the non-ischemic site was measured by ICP-OES.

### Immunohistochemistry and histology

On days 3, 7, 14, and 21 postoperatively, to quantify the necrotic and regenerative muscle fibers, mouse calf muscles were processed into sections of 4 mm thickness and stained with H&E. For immunostaining, the slides were subjected to the antigen retrieval in a sodium citrate solution (pH 6.0) and blocked with 1% bovine serum albumin in PBS containing Tween 20 (Cas# 9006-64-5, Sigma-Aldrich) for 1 h at room temperature. Next, we incubated the tissues with anti-CD31(1:200, Cat# ab222783, Abcam, UK), anti α-SMA antibody (1:500, Cat# GB13044, Servicebio, China), anti-Ki67 antibody (1:1000, Cat# MA5-14520, Invitrogen, USA), and anti-NR4A2 antibody (1:500, Cat# 10975-2-AP, Proteintech, USA) at 4 °C overnight and incubated with secondary antibodies (1:1000; Cat# A11012, Cat# 11,001; Invitrogen) for 2 h at room temperature. Fluorescence microscope images were captured, and the positive areas were counted using the ImageJ software (National Institutes of Health, USA).

### Terminal deoxynucleotidyl transferase dUTP nick end labeling (TUNEL) assay

The TUNEL assay was conducted based on the manufacturer’s protocol for the Click-iT TUNEL assay (Cat# C10245, Invitrogen), and the cells were counterstained with DAPI for 10 min before washing. Images were captured using a fluorescence microscope, and the positive areas were counted using the ImageJ software.

### Dihydroethidium (DHE) staining

DHE (Cat# S0063, Beyotime) staining was used to evaluate ROS generation in the calf muscles. A cutting temperature compound was used to embed calf muscle samples in Tissue-Tek. Then, skeletal muscle cryosections (10 mm) were incubated for 30 min with DHE. Finally, images were obtained using fluorescence microscopy, and DHE-positive areas were counted using ImageJ software.

### Malondialdehyde (MDA) and SOD activity assay

MDA and SOD activity measurements were conducted as directed by the manufacturer for the MDA assay kit (Cat# BC0025, Solarbio, China) and SOD assay kit to detect the MDA and SOD levels of the serum of diabetic mice.

### Cell stimulation

We created HG/Hypo conditions by adding 33.3 mM glucose to the endothelial cell medium with 1% FBS, and the Anaeropack system (Cat# D-07, Mitsubishi Gas Chemical Co, Japan) was used to create a hypoxia condition. Next, HUVECs in a complete endothelial cell medium with 5.5 mM glucose without the Anaeropack system were cultured as the control group (normal glucose, NG). After HUVECs were cultured for 2 days under NG and HG/Hypo conditions, MoS_2_ NDs (50 µg/mL) or PBS were added to the culture medium for 12 h for subsequent experiments. HUVECs were incubated with Baf A1 (1 nM) (Cat# HY-100558, MedChemExpress, USA), 3-MA (Cat#HY-19132, MedChemExpress, USA) and SQ22536 (250 µM) (Cat# S8283, Selleck, USA) to inhibit autophagy and cAMP level, respectively.

### Intracellular ROS and nitric oxide (NO) detection

Intracellular ROS and NO accumulation were determined using the dichlorodihydrofluorescein diacetate (DCFH-DA) probe (Cat# S0033S, Beyotime) and the 3-amino,4-aminomethyl-2’, 7’-fluorescein diacetate (DAF-FM-DA) probe (Cat# S0019, Beyotime). The cells were incubated in 5 µM DCFH-DA or DAM-FM DA for 30 min. The harvested cells were measured by fluorescence intensity using microscopy and flow cytometry.

### Wound healing assay

HUVECs were pretreated until they reached 100% confluence, and an artificial gap was generated using a 200 µL micropipette tip. Images were acquired using inverted microscopy (IX71, Olympus) immediately after the scratches were generated and 24 h later. Finally, the cell migration area was quantified by the closure of the scratch area.

### Transwell assay

HUVECs were seeded onto each Transwell inserts (Cat# 3422, Corning, USA). A culture medium of 500 L was added to the lower chamber. We removed the medium after 12 h and fixed them for 30 min. Subsequently, each well was stained for 20 min with 0.2% crystal violet. After removing the stain, the cells were photographed by the inverted microscope.

### Tube formation

Matrigel (Cat# 356,234, Corning) was added before solidifying at 37 °C for 20 min, and the cell solution (100 µL) with 2 × 10^4^ cell numbers was added to the gel and incubated for 4 h. Next, the tubes were photographed using an inverted microscope, and the vessel length and number were counted using the ImageJ software.

### Immunofluorescence staining

We seeded HUVECs on 24-well plates, fixed them with 4% PFA, and incubated them with anti-p16 antibodies (1:200, Cat# 10883-1-AP, Proteintech), anti-p21 antibody (1:200, Cat# 2947, Cell Signaling Technology, USA), anti-Lamp1 antibody (1:200, Cat# 9091, Cell Signaling Technology), and anti-p62 antibody (1:200, Cat# 88,588, Cell Signaling Technology) overnight at 4 °C and secondary antibodies (1:1000; Cat# A11012, Cat# 11,001; Invitrogen) at room temperature, with DAPI staining nuclei for 10 min, and the cells were taken by inverted fluorescence microscopy.

### Annexin V-FITC/propidium iodide (PI) assay

An annexin V-FITC/PI assay kit (Cat# 521942, Solarbio) was used for this experiment. Following pretreatment with Annexin V-FITC in the dark for 20 min, HUVECs are resuspended in the binding buffer and analyzed by flow cytometry.

### 5-Ethynyl-2-deoxyuridine (EdU) assay

HUVECs were incubated with 10 µM EdU (Cat# C10310, Riobobio, China) for 6 h, with a fixation for 30 min and a permeabilization for 20 min (0.5% Triton X-100) before the Click-iT reaction cocktail (Cat# C10310-1, Riobobio) for 45 min at room temperature. A DAPI counterstain was applied to the cells, which were photographed using fluorescence microscopy, and EdU^+^ cells were counted using the ImageJ software.

### Cell transfection

Autophagy activation was measured using lenti-mRFP-GFP-LC3 (Cat# GM-1314l204H, Genomeditech, China). Autolysosomes are indicated in the presence of co-localized GFP and RFP fluorescence, and autolysosomes are indicated in the absence of GFP. Transfection efficiency was determined using fluorescence microscopy and laser scanning confocal microscopy (LSCM) (Sted 8; Leica, Germany).

NR4A2 small interfering RNA was obtained from Genomeditech (Shanghai, China). The sequences are as follows:

5’-GGACAGCAGUCCUCCAUUATTUAAUGGAGGACUGCUGUCCTT-3’.

Si-*Ctrl* or si-*NR4A2* was transfected with 50 nM using lipofectamine3000 (Cat# L3000075; Invitrogen), as per the manufacturer’s instructions. The effects of NR4A2 knockdown were also assessed by western blotting.

### Cell cycle assay

After the pretreatment, cells were collected with a fixation in 70% ethanol at 4 °C overnight, followed by staining with PI (Cat# 1052, Beyotime), staining solution in the dark for 30 min, and performing flow cytometry measurement. We determined the percentage of cells in the G1, S, and G2 phases using FlowJo software (BD Biosciences, USA).

### 5,5’,6,6’-Tetrachloro-1,1’,3,3’-tetraethylbenzimidazolylcarbocyanine iodide (JC-1) staining

Cells were collected for early apoptosis detection based on potential changes in the mitochondrial membrane measured using a mitochondrial membrane potential kit (Cat# C2003S, Beyotime) and fluorescence microscopy.

### Western blotting

After pretreatment, HUVECs were lysed to obtain proteins, which were separated at equal loadings using a running buffer (18.80 g Glycine, 3.02 g Tris, and 1 g SDS). A polyvinylidene difluoride membrane (Cat# IPVH00010; Millipore, USA) was used to transfer the samples. Next, the membrane was incubated with anti-p16 (1:1000), anti-p21 (1:1000), anti-Bax (1:1000, Cat# 2774, Cell Signaling Technology, Danvers, MA, USA), anti-Bcl-2 (1:1000, Cat# 3498, Cell Signaling Technology), anti-cleaved caspase-3 (1:1000, Cat# 9664, Cell Signaling Technology), anti-LC3 (1:1000, Cat# 12741, Cell Signaling Technology), anti-p62 antibody (1:1000), anti-Lamp1 antibody (1:1000), anti-NR4A2 antibody(1:1000) anti-cAMP antibody(1:2000, Cat# ab76238, Abcam), anti-PKA antibody(1:1000, Cat# 4782, Cell Signaling Technology), anti-p-PKA antibody (1:1000, Cat# 5661, Cell Signaling Technology), anti-ATG4B antibody (1:1000, Cat# A5059, ABclone), anti-ATG9B antibody (1:1000, Cat# A7406, ABclone), anti-ATG13 antibody (1:1000, Cat# A0690, ABclone), anti-VEGFR2 antibody (1:1000, Cat#2479, Cell Signaling Technology), anti-PI3K antibody (1:1000, Cat#4249, Cell Signaling Technology), anti-p-AKT antibody (1:1000, Cat#4046, Cell Signaling Technology), anti-t-AKT antibody (1:1000, Cat#4691, Cell Signaling Technology), anti-p-eNOS antibody (1:1000, Cat#9571, Cell Signaling Technology), and anti-t-eNOS antibody (1:1000, Cat#9572, Cell Signaling Technology). Horseradish peroxidase-conjugated secondary antibodies (1:10000; Cat# ab6721, Cat# ab4728; Abcam) were subsequently incubated for 2 h and detected using an electrochemiluminescence system. Anti-GAPDH (1:10000, Cat# 60004-1-lg, Proteintech) and anti-β-Actin antibody (1:10000, Cat# 20536-1-AP, Proteintech) were used as internal references.

### Quantitative reverse transcription polymerase chain reaction (RT-qPCR)

RT-qPCR was performed to determine the expression of NR4A2, FOXM1, SOX6, and ZMIZ1 Briefly, and total RNA was isolated from HUVECs using TRIzol Reagent (Cat# 15596026, Invitrogen). The extracted RNA was reverse-transcribed (Cat# RR036A, Takara, Japan). RT-qPCR was conducted using a TB Green RT-qPCR Kit (Cat# RR820A, Takara) to determine the expression of the target genes. All primers used are listed in Table S3.

### Transcriptome analysis

Total RNA was extracted from HUVECs after pretreatment and harvesting. A Qubit Fluorometer (Qubit Flex, Thermo Fisher Scientific) and Tapestation (4200 Tapastation, Agilent) were used to measure the RNA quality and quantity. Sequencing libraries were prepared using the SMARTer Universal Low Input RNA Kit (Takara). Mammalian-specific R-probes were used to cleave ribosomal cDNA using ZapR. PCR primers matching the Illumina adapters were used to enrich the remaining fragments. The libraries were normalized to 10 nM Tris-HCl (10 mM, pH 8.5) containing 0.1% Tween 20. To map and trim the FASTQ format, A TrimGalore sequence was generated and a FastQC-based quality assessment was conducted on the sequences. A Hisat2 aligner was used for alignment with the Ensembl reference genome, GRCm38. Gene expression was calculated using the R package ‘feature counts’ (R Foundation, New Zealand).

Before the analysis, we used a cutoff of 1 to define the minimum level of expression for each gene, and a negative binomial model was implemented in the Bioconductor package DESeq to calculate differential gene expressions. Significantly differentially expressed genes were defined as having *P* < 0.05, and pathway analysis was performed using the Cluster Profiler R package.

### Chromatin immunoprecipitation sequencing (ChIP-seq) analysis

ChIP-seq data for NR4A2 were downloaded from the Gene Expression Omnibus database (GSE186197). The R package “ChIPseeker” was used to depict the positions of NR4A2-binding peaks on genome-wide scales. The sequenced reads were mapped to the human genome (hg38) using Integrative Genomics Viewer. *De novo* DNA motif analysis and construction from the ChIP-seq data were performed using MEChIP.

### Statistical analysis

The mean and standard deviations of all data were calculated using GraphPad Prism version 9.0 (GraphPad Software, USA). One-way analysis of variance (ANOVA), two-way ANOVA, and *t*-test were used for comparisons, and signs of significance were defined as *P* < 0.05.

## Results and discussion

### MoS_2_ NDs preparation and characterization

**Fig. 2:**
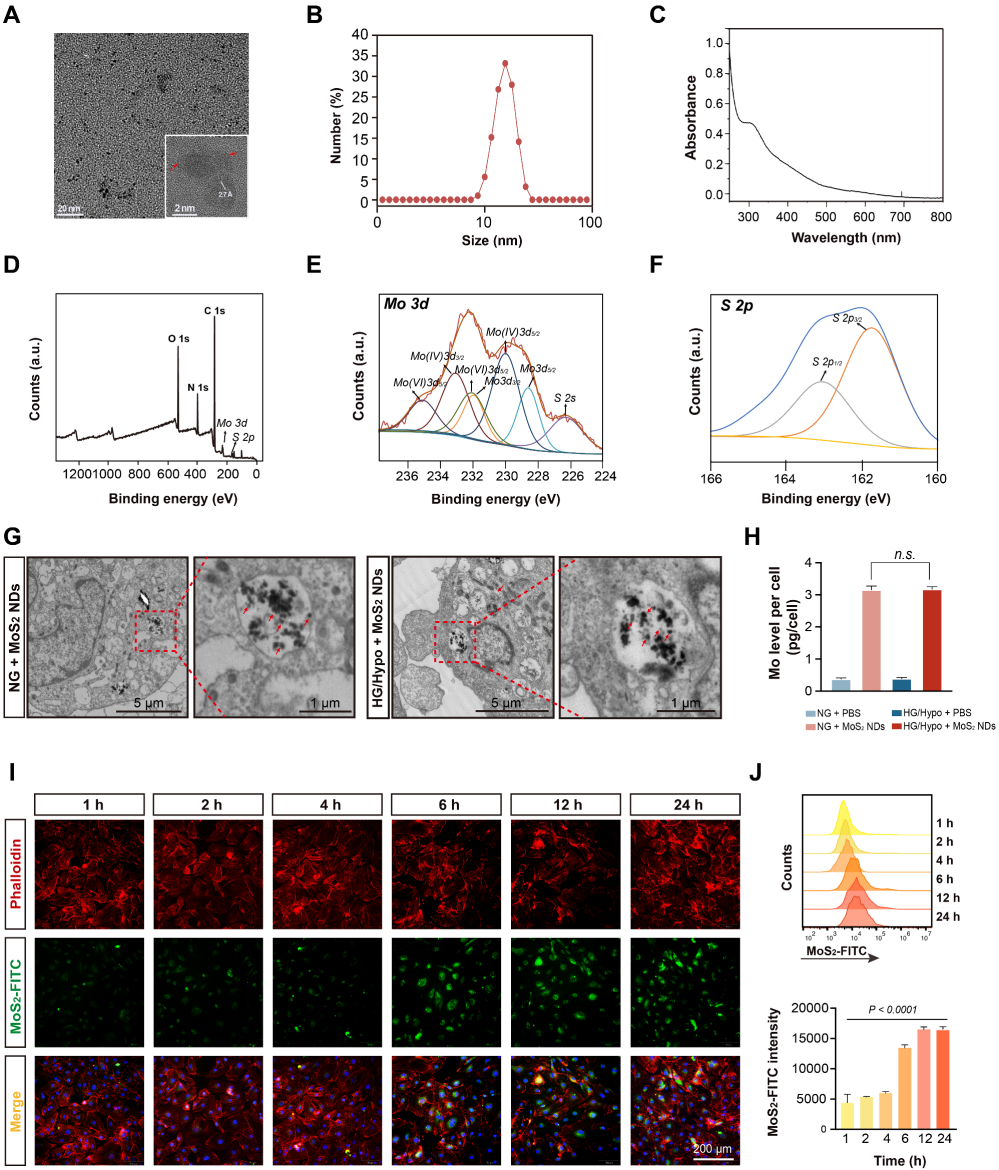
Characterization of synthesized MoS_2_NDs (**A**) TEM image of MoS_2_ NDs, inset shows HRTEM image of MoS_2_ NDs. The red arrows indicate the boundary around the clear lattice fringe. (**B**) Size distribution of MoS_2_ NDs in PBS. (**C**) The UV to vis absorption spectra of MoS_2_ NDs. (**D**) XPS data showing the survey spectrum and (**E**) spectrum of Mo 3d and (**F**) spectrum of S 2p. (**G**) Representative images of bio-TEM of MoS_2_ NDs within HUVECs under NG conditions and HG/Hypo conditions. (**H**) The Mo level in HUVECs under NG conditions and HG/Hypo conditions, *n* = 3 per group. P values were calculated by two-way ANOVA with Tukey’s post hoc test. (**I**) Representative images of intracellular uptake of MoS_2_ NDs from different time points using fluorescence microscopy. (**J**) Intracellular uptake of MoS_2_ NDs by flow cytometry and quantification of fluorescence intensity of intracellular MoS_2_ NDs, *n* = 3, and P values were calculated by one-way ANOVA. All data are expressed as mean ± SD.

MoS_2_ NDs were developed using a one-step solvothermal process, wherein ammonium tetrathiomolybdate [(NH_4_)_2_MoS_4_] was dissolved in a reducing environment (hydrazine hydrate) at 120 °C for 3 h (Fig. 1) [35]. To control the particle size and stabilize the MoS_2_ ND, PVP was introduced as a dispersion stabilizer during the preparation process. TEM images showed that the MoS_2_ NDs exhibited a uniform sphere shape with a diameter of approximately 2.2 nm (Fig. 2A). The high-resolution TEM (HRTEM) image (inset) of MoS_2_ NDs illustrated the hexagonal lattice structure with a lattice space of 2.7 Å, which was assigned to the (100) planes of the typical hexagonal structure of MoS_2_ [43]. The XRD pattern also demonstrated the good crystallinity of MoS_2_ NDs (Figure S1). Additionally, they dispersed well in water with a hydrodynamic size of 13.5 ± 2.3 nm (Fig. 2B) and a zeta potential of -16.5 mV. The size difference of the MoS_2_ NDs determined by dynamic light scattering (DLS) and TEM mainly resulted from the absorption of water molecules since DLS performed wet samples [44]. Notably, only when the hydrodynamic diameter of the nanoparticles is smaller than the glomerular filtration threshold (20 nm) of the kidney can nanomaterials be excreted through the renal clearance pathway, which exhibits exceptional biosecurity [45–47]. MoS_2_ NDs have also been demonstrated to have such potential, as reported by earlier investigations [35, 36], which was consistent with our results. The UV–vis absorption spectra of the MoS_2_ NDs are shown in Fig. 2C. Furthermore, the compositions of MoS_2_ NDs were measured by XPS. The characteristic binding peaked at 228.6 eV, 230.0 eV, and 231.9 eV, which corresponded to the 3d_3/2_ and 3d_5/2_ peaks of Mo^4+^, respectively, and characteristic peaks located at 232.0 and 235.1 eV could be ascribed to the Mo^6+^. Moreover, the 161.7 eV and 163.4 eV peaks could be assigned to the S 2p_3/2_ and 2p_1/2_ (S^2-^), respectively (Fig. 2D–F). Also, the strong 3d_3/2_ and 3d_5/2_ peaks of Mo (230.0, 232.0, 233.1, and 235.1 eV) and the peak at 530.37 eV of O indicated the formation of the Mo-O bond (Figure S2, Table S1). These data demonstrate that Mo in the MoS_2_ NDs had reduced (IV) and oxidized (VI) states, enabling it to perform redox interactions, which is consistent with previous studies [35, 48]. These data confirmed the successful MoS_2_ NDs synthesis. Furthermore, MoS_2_ NDs were dispersed in three media: deionized water (DI water), phosphate-buffered saline (PBS), and Dulbecco’s modified Eagle’s medium (DMEM), and stabilized after storing for at least a week, indicating their stability under physiological conditions (Figure S3).

As shown in Fig. 2G, bio-TEM revealed a large number of nanodots in the endothelial cells without significant damage, degradation, or deformation under normal glucose (NG) conditions. Moreover, by ICP-OES, the level of Mo dissolution was measured in endothelial cells. After 24 h incubation, the Mo level increased from 0.37 ± 0.03 pg/cell to 3.15 ± 0.12 pg/cell (Fig. 2H). This indicated that MoS_2_ NDs could enter into endothelial cells and exhibited good stability within endothelial cells. Furthermore, to evaluate the in vitro uptake efficiency, FITC-labeled MoS_2_ NDs were prepared by mixing MoS_2_ NDs with FITC-PEG-SH under sonication and incubating at room temperature. Fourier-transform infrared spectroscopy (FT-IR) spectra confirmed the successful preparation of FITC-labeled MoS_2_ NDs (Figure S4). Next, the FITC-labeled MoS_2_ NDs were incubated with HUVECs for 1, 2, 4, 6, 12, and 24 h. Fluorescence microscopy revealed that the FITC signal intensity increased over time and remained constant after 12 h (Fig. 2I). Moreover, the fluorescence intensity measured using flow cytometry was consistent with fluorescence microscopy (Fig. 2J), which indicated that the uptake of MoS_2_ NDs by endothelial cells depended on the incubation duration.

Subsequently, in vivo biodistribution of MoS_2_ NDs was measured in healthy mice. The liver and spleen exhibited the highest dose distributions, whereas the heart and lungs exhibited the lowest distribution (Figure S5A). Moreover, MoS_2_ NDs rapidly accumulated in the kidney within 6 h and were subsequently excreted through the urine within 24 h, thereby indicating the effective renal excretion of the MoS_2_ NDs. Next, the in vivo metabolism of MoS_2_ NDs was examined by evaluating the Mo content in feces and urine at days post-administration. It was found that the feces and urine showed the highest Mo levels at days post-administration, which decreased over time (Figure S5B). We also characterized the morphology of MoS_2_ NDs in feces and urine by TEM, confirming that MoS_2_ NDs existed in feces and urine as their original form, further demonstrating the excellent metabolic capacity of MoS_2_ NDs (Figure S6A-B). The biosafety of the MoS_2_ NDs was confirmed using the CCK-8 assay on HUVECs (Figure S7), which showed no cytotoxicity from 25 to 200 µg/mL. No significant differences in the indexes of VCAM-1 and ICAM-1 in endothelial cells, as well as IFN-γ, IL-1β, IL-6, and TNF-α in healthy mice serum between the MoS_2_ NDs and PBS-treated was noted, indicating MoS_2_ NDs did not cause inflammatory responses both in vitro and in vivo (Figure S8-S9). Major organ histopathology showed no fibrosis, necrosis, or no hemorrhage in the lungs, livers, spleens, kidneys, or hearts 7 days after treatment with MoS_2_ NDs (Figure S10). Furthermore, routine blood parameters (Figure S11A-D) and liver and kidney function (Figure S11E-F) were not significantly different between mice injected with PBS and MoS_2_ NDs. The hemolysis rate of MoS_2_ NDs at different concentrations was determined by using red blood cells (Figure S12). No obvious red color was observed even at the high concentration of 100 µg/mL–500 µg/mL (hemolysis rate < 5%), indicating the good blood compatibility of MoS_2_ NDs. These results demonstrate the biosafety of MoS_2_ NDs in vivo. These results also confirmed that the particle size and hydrodynamic diameter of MoS_2_ NDs were sufficient for their metabolism through the kidney while exhibiting exceptional biosafety both in vivo and in vitro.

Subsequent experiments in this study focused on applying MoS_2_ NDs in diabetic contexts, which led to oxidative stress [49] and a decrease in the pH level in the cytoplasm [50]. Therefore, we checked the stability of MoS_2_ NDs in acidic and oxidizing environments (pH 5.0, 200 mM H_2_O_2_). There were no significant changes in the diameter distribution and absorption spectra after storage for 7 days, while a measure of biodegradation after 7 days of co-incubation (Figure S13). Additionally, the TEM image indicated that the structure and morphology of MoS_2_ NDs remained intact integrally but showed slight structural changes on day 7 (Figure S14), thereby demonstrating the acceptable stability of the designed MoS_2_ NDs in acidic and oxidizing environments. Moreover, HG/Hypo conditions did not affect the uptake behavior of MoS_2_ NDs by endothelial cells (Fig. 2H), and bio-TEM also showed intact MoS_2_ NDs within endothelial cells under HG/Hypo conditions (Fig. 2G). These results illustrated the stability of MoS_2_ NDs and laid the foundation for their application in diabetes settings.

### The antioxidative properties of the MoS_2_ NDs

**Fig. 3:**
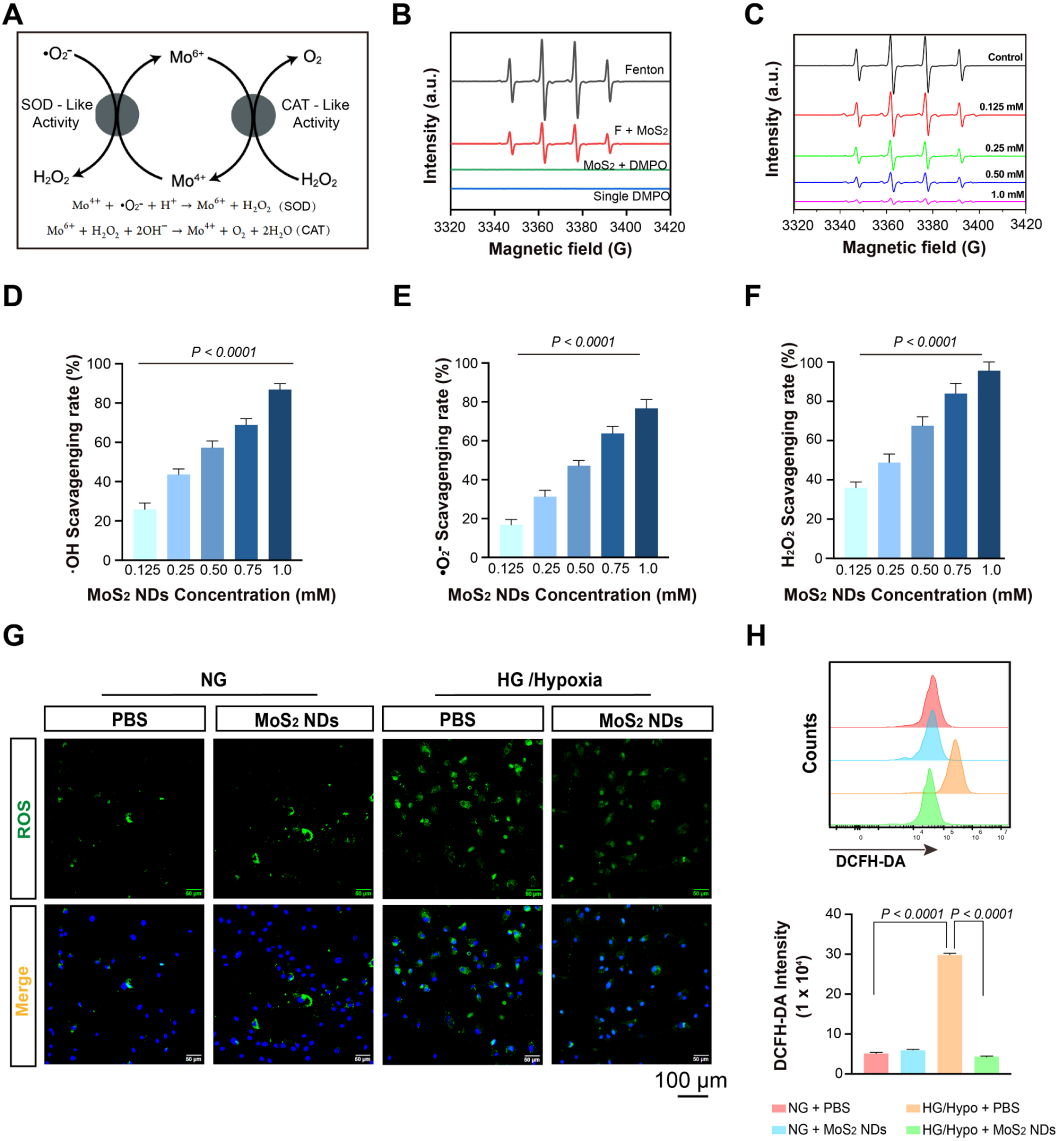
ROS scavenging ability of MoS_2_NDs (**A**) Schematic illustration of enzyme-mimicking activities of MoS_2_ NDs. (**B**) ESR spectra of DMPO/•OH adducts obtained from samples with Fenton, Fenton and MoS_2_ NDs, MoS_2_ NDs and DMPO, and single DMPO. (**C**) ESR spectra of DMPO/•OH adducts obtained from different concentrations of MoS_2_ NDs. The ROS scavenging capacity of MoS_2_ NDs, including exterminating (**D**) •OH, (**E**) •O_2_^-^, and (**F**) H_2_O_2_ (50 mM) at different concentrations of Mo, *n* = 3. P values were calculated by one-way ANOVA. (**G**) Representative images of HUVECs with intracellular ROS probe, DCFH-DA, from different treatments. (**H**) DCFH-DA fluorescence by flow cytometry and quantification of DCFH-DA fluorescence intensity, *n* = 5, each group, and P values were calculated by two-way ANOVA with Tukey’s post hoc test. All data are expressed as mean ± SD.

Reportedly, MoS_2_-based nanomaterials possess ROS-scavenging capacity owing to their intrinsic enzyme-like activities (e.g., SOD, CAT, and peroxidase) [34, 36]. The possible mechanisms of the enzyme-mimicking properties involve switching the Mo (IV)/Mo (VI) redox couple on the surface of MoS_2_ [48], where the Mo (VI) sites are responsible for the H_2_O_2_ oxidation through CAT mimetics and Mo (IV) sites are used to scavenge •O_2_^-^ and •OH (Fig. 3A). The •OH free radicals were determined with DMPO spin traps monitoring by EMX1598 ESR spectra. A distinct signal for the DMPO/•OH adducts was induced by the Fe^2+^/H_2_O_2_ system (Fig. 3B). MoS_2_ NDs effectively scavenged •OH, as indicated by the reduced signal intensity of DMPO/•OH, demonstrating the •OH scavenging capacity of the MoS_2_ NDs (Fig. 3C). Additionally, the clearance ratio of •OH was dose-dependent, as determined by the •OH-specific probe orthophenanthroline (Fig. 3D). We also monitored the superoxide anion-decomposition ability of the MoS_2_ NDs using the SOD assay (Fig. 3E). The scavenging rate increased from 16.8 to 76.7% at different concentrations, confirming that MoS_2_ NDs exhibited SOD-like activity to quench •O_2_^-^. In addition, the CAT-like capacity of MoS_2_ NDs was determined by the H_2_O_2_-specific probe xylenol orange. As shown in Fig. 3F, there was a significant increase in the clearance ratio of H_2_O_2_, sharply increasing from 35.9 to 95.2% with increasing concentrations of MoS_2_ NDs. Meanwhile, visible bubbles (O_2_) were produced in the presence of MoS_2_ NDs and H_2_O_2_, which further indicated the CAT-like activity of MoS_2_ NDs (Figure S15). Also, the XPS spectra of MoS_2_ NDs were measured after reacting with H_2_O_2_ to reflect the valence state changes (Figure S16). After the treatment with H_2_O_2_ (50 mM), the peaks at 228.5 eV assigned to Mo 3d5/2 of Mo^5+^ were shifted to higher intensities, and the Mo 3d5/2 and Mo 3d3/2 peaks at 231.8 and 234.8 eV of Mo^6+^ were shifted to lower intensities, suggests the valence change of molybdenum element after the addition of H_2_O_2_. Overall, MoS_2_ NDs exhibited robust CAT, SOD­like activities, and •OH scavenging ability; they were used as an antioxidant for subsequent evaluation. Excessive ROS are generated under diabetes conditions [51, 52]. Therefore, the intracellular ROS probe, DCFH-DA, was used to detect the ROS content in endothelial cells *via* fluorescence microscopy and flow cytometry. Although MoS_2_ NDs did not alter the ROS level of endothelial cells under NG conditions, they significantly reduced the intracellular ROS content to basal levels under HG/Hypo conditions (HG/Hypo + PBS = 29.78 ± 0.40 × 10^4^ vs. HG/Hypo + MoS_2_ NDs = 4.35 ± 0.12 × 10^4^, *P* < 0.0001) (Fig. 3G-H), thereby confirming MoS_2_ NDs as an effective intracellular ROS scavenger in diabetic context.

Overall, the conducted experiments have highlighted the outstanding biosafety, robust stability, remarkable efficiency in cellular uptake, and significant ROS scavenging ability of MoS_2_ NDs. This not only distinguishes them from other forms of MoS_2_ but also underscores their advantages compared with all other nanomaterials (such as silver nanoparticles, zinc oxide nanoparticles, and nanoceria) utilized in the treatment of diabetes-related complications.

### MoS_2_ NDs improved collateral formation in diabetic mice

In the hindlimb ischemia model, the femoral arteries of mice are ligated. This process causes an increase in angiogenetic factors in the ischemic muscle [53]. As a result, endothelial cells may be activated and mobilized, forming new leaky blood vessels, leading to the accumulation of nanoparticles caused by the enhanced permeability and retention (EPR) effect [54]. We also further demonstrated the passive targeting of MoS_2_ NDs owing to the EPR effect at ischemic muscles by in vivo fluorescence imaging. Cy5.5-MoS_2_ NDs were injected through the tail vein, and the fluorescence signal increased with time. At 3–6 h after injection, the signal reached the peak, which decreased later owing to in vivo metabolism (Figure S17A). The fluorescence intensity was stronger at the ischemic site than at the non-ischemic site (Figure S17A). In addition, the CleVue Basic system was used to perform a semi-quantitative analysis of the fluorescence intensity of Cy5.5 at the ischemic site, which further validated the aforementioned findings (Figure S17B). Moreover, in vivo biodistribution of MoS_2_ NDs in the ischemic site and the non-ischemic site was measured to further confirm the EPR effect at ischemic muscles (Figure S18). Therefore, MoS_2_ NDs were shown to be targeted to the ischemic site, thus laying the groundwork for subsequent therapeutic studies in vivo.

**Fig. 4:**
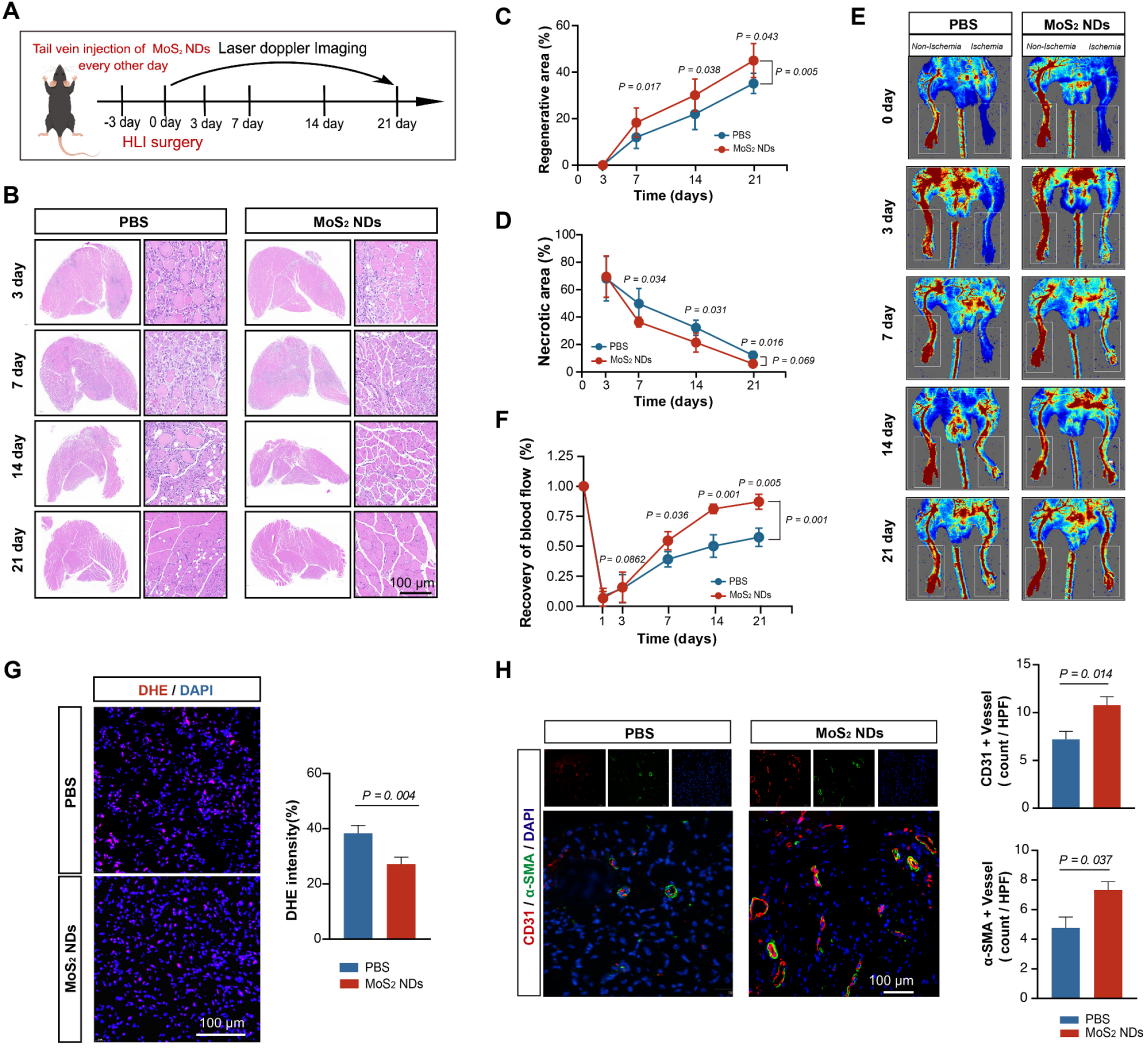
MoS_2_ NDs improved collateral formation in diabetic mice. (**A**) Illustration of time points of MoS_2_ NDs injection and laser Doppler imaging in diabetic mice. (**B**) Representative H&E images of muscles harvested at the indicated time with PBS and MoS_2_ NDs. (**C**) Quantification of the regenerating area at different time points, *n* = 5, and P values were calculated by two-way ANOVA with Bonferroni post-hoc test. (**D**) Quantification of the necrotic area at indicated times from H&E staining, *n* = 5, and P values were calculated by two-way ANOVA with Bonferroni post-hoc test. (**E**) Representative images of hindlimb blood perfusion with PBS and MoS_2_ NDs at indicated times. (**F**) Quantification of hindlimb blood perfusion at the indicated times, *n* = 5, and P values were calculated by two-way ANOVA with Bonferroni post-hoc test. (**G**) Representative DHE staining images and quantification of DHE^+^ area on day 7, *n* = 9, P values were calculated by *t*-test. (**H**) Representative CD31^+^/α-SMA^+^ immunofluorescent images and quantification of CD31^+^ area and α-SMA area on transverse cross sections on day 7, *n* = 9, and P values were calculated by *t*-test. All data are presented as the mean ± SD.

Subsequently, to evaluate the effect of MoS_2_ NDs on the restoration of blood flow and neovessels formation after HLI in HFD/STZ-induced type 2 diabetic mice, we treated the mice with PBS or MoS_2_ NDs every other day from 3 days preoperatively to 21 days postoperatively (Fig. 4A). Although no changes were observed in blood glucose change (Table S2), the MoS_2_ NDs group exhibited superior recovery, as indicated by the faster increase of muscle regeneration (Area under Curve (AUC) _[MoS2 NDs]_ = 4.68 ± 0.50 vs. AUC_[PBS]_ = 3.43 ± 0.41, *P* < 0.001), faster reduction of muscle necrosis (AUC _[MoS2 NDs]_ = 5.10 ± 0.49 vs. AUC_[PBS]_ = 6.78 ± 0.63, *P* < 0.001) (Fig. 4B-D, Figure S19A-B), and faster blood flow recovery (AUC _[MoS2 NDs]_ = 15.06 ± 0.70 vs. AUC_[PBS]_ = 10.76 ± 0.77, *P* < 0.001) after HLI surgery (Fig. 4E-F, Figure S19C).

The area of fibrosis was also significantly reduced, as indicated by Masson staining (Figure S20). Furthermore, the ischemia score at the aforementioned time points also revealed that MoS_2_ NDs improved the blood supply (Figure S21). Next, the ROS scavenging ability of MoS_2_ NDs was also confirmed in vivo, as indicated by the reduced DHE intensity in the muscle sections in the MoS_2_ NDs group (Fig. 4G), as well as the decreased MDA level and increased SOD level in the serum of diabetic mice (Figure S22). The data showed that MoS_2_ NDs had a positive impact on reducing oxidative stress in diabetic settings. Additionally, the number of CD31^+^ and α-SMA^+^ vessels was significantly higher in the MoS_2_ NDs group (Fig. 4H), indicating increased angiogenesis post-HLI in the MoS_2_ NDs group. These findings provided strong evidence of MoS_2_ NDs improving blood flow restoration and neovessel formation at the ischemic site after HLI in diabetic mice.

### MoS_2_ NDs improved endothelial cell angiogenic dysfunction induced by high glucose and hypoxia

**Fig. 5:**
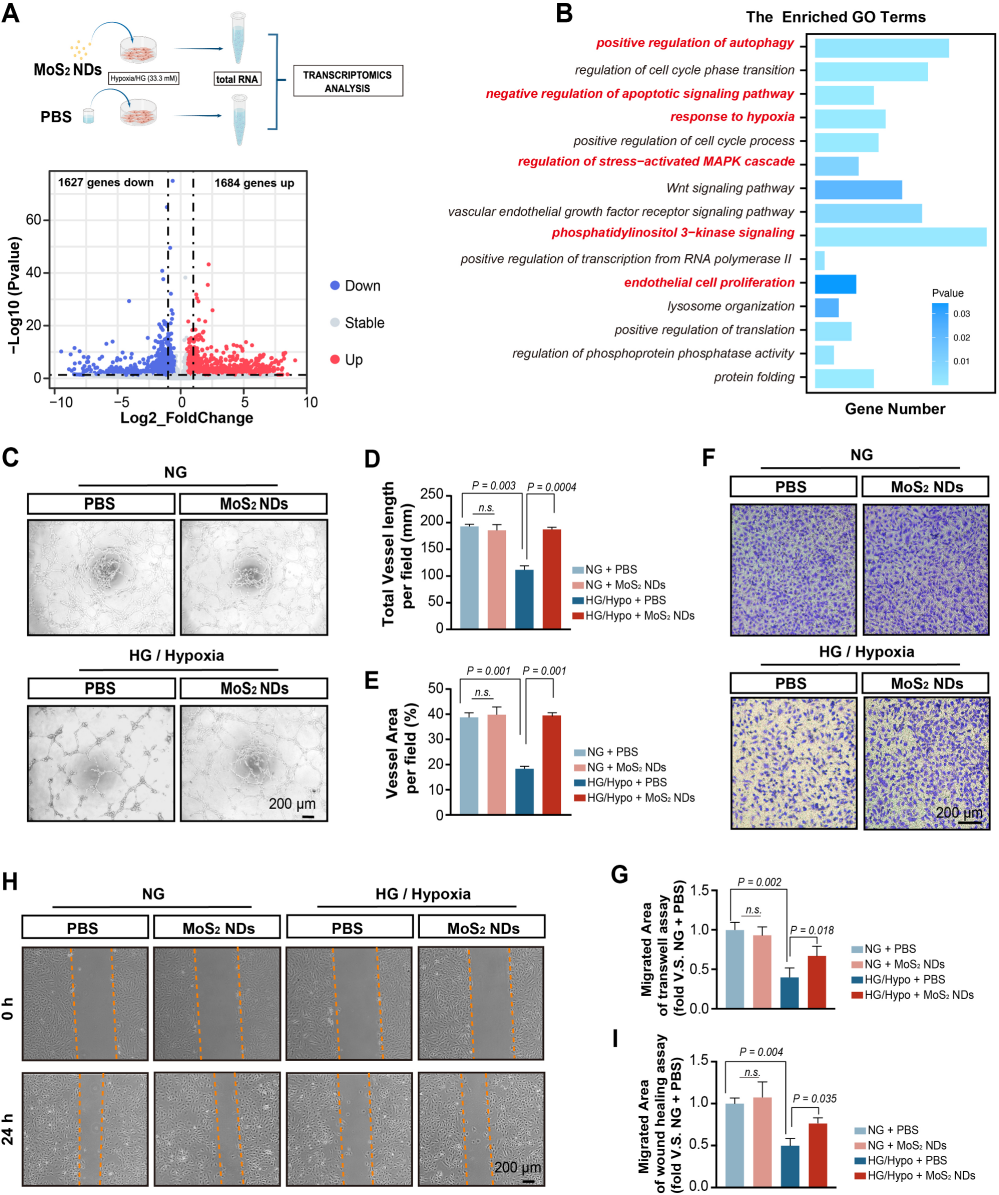
MoS_2_NDs alleviated endothelial cell angiogenic dysfunction induced by HG/Hypo. (**A**) Illustration of transcriptomics of HUVECs treated with MoS_2_ NDs and PBS under HG/Hypo conditions. (**B**) Pathways enriched by differentially expressed genes using GO enrichment analysis. (**C**) Representative tube formation images of HUVECs with NG + PBS, NG + MoS_2_ NDs, HG/Hypo + PBS, and HG/Hypo + MoS_2_ NDs. (**D**) Total vessel length per field, and (**E**) vessel area per field, *n* = 4, and P values were calculated by two-way ANOVA with Tukey’s post hoc test. (**F**) Transwell assay of HUVECs and (**G**) cell migration rate with different treatments, *n* = 4, and P values were calculated by two-way ANOVA with Tukey’s post hoc test. (**H**) Wound healing assay of HUVECs and (**I**) cell migration rate with different treatments, *n* = 4, and P values were calculated by two-way ANOVA with Tukey’s post hoc test. All data are expressed as mean ± SD.

Endothelial cells are determinants for the maintenance of vascular integrity and blood flow. During collateral formation, endothelial cells recruit pericytes, parietal cells, smooth muscle cells, and factors to form blood vessels [55, 56]. Therefore, restoring endothelial function and promoting neovascularization are effective strategies for treating diabetic vascular diseases and have become the focus of current research [57, 58]. Therefore, when MoS_2_ NDs have gained therapeutic benefit in vivo, we focused our research on endothelial cells to explore the specific mechanism of how MoS_2_ NDs alleviate endothelial dysfunction and thus promote angiogenesis. RNA sequencing analysis was conducted under HG/Hypo conditions in endothelial cells to identify the potential mechanisms of angiogenesis regulated by MoS_2_ NDs. A total of 3311 genes were differentially expressed, 1684 of which were upregulated and 1627 were downregulated (Fig. 5A). These genes were enriched for multiple angiogenesis pathways such as PI3K-AKT [59], MAPK signaling pathways [60], and endothelial proliferation by Gene Ontology (GO) enrichment analysis and Kyoto Encyclopedia of Genes and Genomes (KEGG) enrichment analysis (Fig. 5B, Figure S23-S26).

To confirm the pro-angiogenic effects of MoS_2_ NDs in vitro, we conducted tube formation, wound healing, and transwell assays. Tube formation showed that, under NG conditions, the total vessel length and vessel area per field between the PBS and MoS_2_ NDs remained unchanged. In contrast, under HG/Hypo conditions, the total vessel length and vessel area significantly increased with the administration of MoS_2_ NDs (Fig. 5C-E). Additionally, transwell and wound healing assays revealed that the migration area increased significantly with MoS_2_ NDs administration under HG/Hypo conditions (Fig. 5F-I). Moreover, the intracellular nitric oxide (NO), a pro-angiogenic factor, was detected by DAF-FM-DA and showed increased amounts of NO upon treatment with MoS_2_ NDs under HG/Hypo conditions (Figure S27). All the above data demonstrated the pro-angiogenic effects of MoS_2_ NDs under HG/Hypo conditions in vitro.

### MoS_2_ NDs inhibited cell apoptosis induced by HG/Hypo and promoted endothelial cell proliferation

**Fig. 6:**
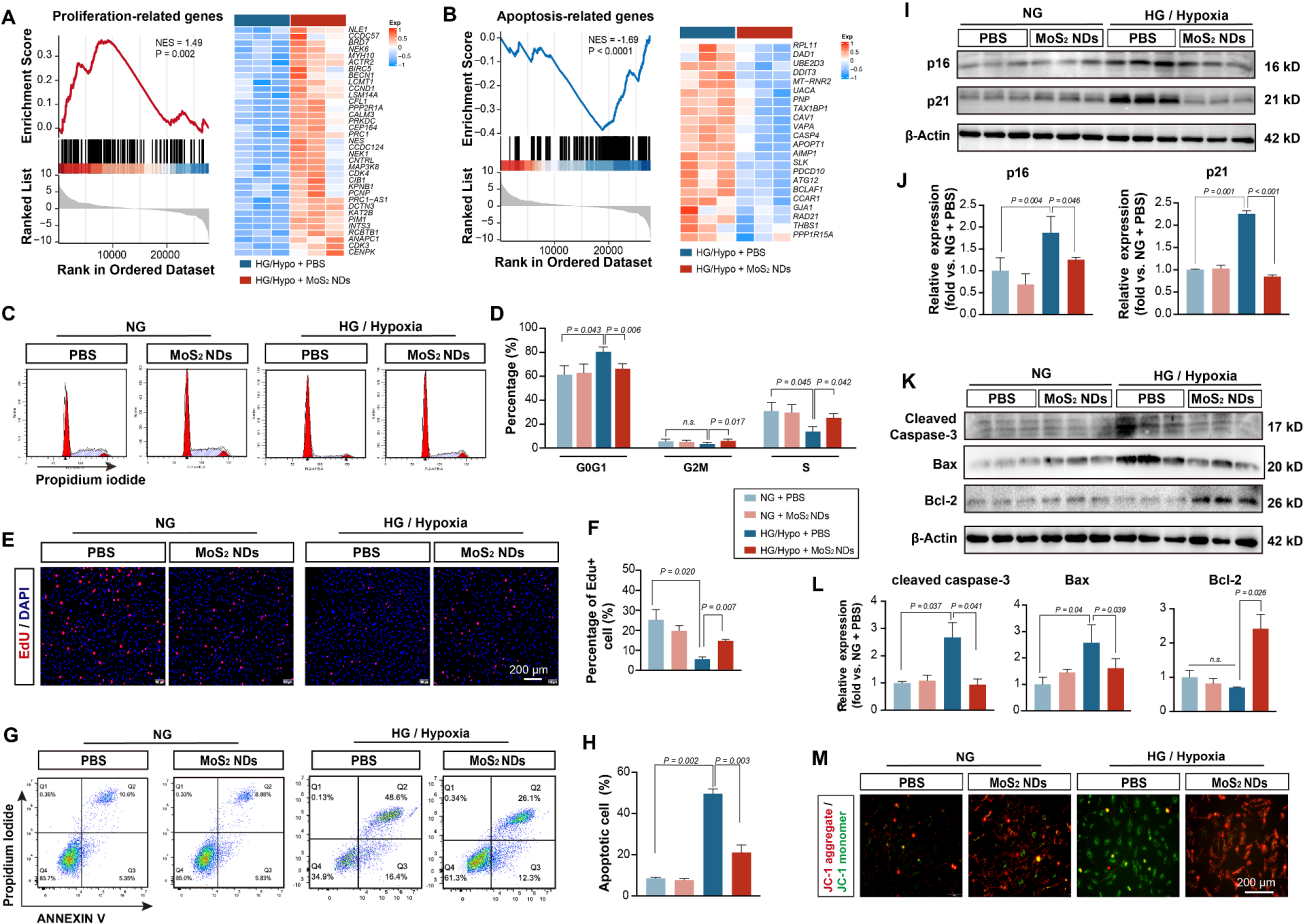
MoS_2_NDs inhibited cell apoptosis induced by HG/Hypo and promoted endothelial cell proliferation. (**A**) GSEA plot and heatmap of proliferation-associated genes. (**B**) GSEA plot and heatmap related to apoptosis. (**B**) Cell cycle analysis using flow cytometry with different treatments. (**D**) Percentage of HUVECs on different cell phases, *n* = 3, and P values were calculated by two-way ANOVA with Tukey’s post hoc test. (**E**) EdU staining on HUVECs and (**F**) percentage of EdU^+^ cells, *n* = 4, and P values were calculated by two-way ANOVA with Tukey’s post hoc test. (**G**) Annexin V/PI flow cytometric detection with different treatments. (**H**) Percentage of apoptotic cells with different treatments, *n* = 3, and P values were calculated by two-way ANOVA with Tukey’s post hoc test. (**I**) p16 and p21 expressions on HUVECs and (**J**) quantification of p16 and p21 protein levels with different treatments, *n* = 3, and P values were calculated by two-way ANOVA with Tukey’s post hoc test. (**K**) The expressions of apoptosis-associated factors: cleaved caspase-3, Bax, Bcl-2, and (**L**) quantification of these protein expression levels with different treatments, *n* = 3, and P values were calculated by two-way ANOVA with Tukey’s post hoc test. (**M**) Representative images of JC-1 staining showing early apoptosis of HUVECs with different treatments. All data are expressed as mean ± SD.

Endothelial proliferation is important for endothelial angiogenesis. Impaired angiogenesis can be attributed to decreased cell proliferation levels and increased apoptosis levels of endothelial cells under HG/Hypo conditions [61, 62]. To determine whether MoS_2_ NDs could promote endothelial proliferation and inhibit endothelial apoptosis induced by HG/Hypo conditions, Gene Set Enrichment Analysis (GSEA) was performed. As shown in Fig. 6A and B, the GSEA and heatmaps indicated positive enrichment associated with proliferation and negative enrichment associated with apoptosis. Next, a set of experiments was conducted to confirm the effect of MoS_2_ NDs on the proliferation and apoptosis of endothelial cells. We conducted a cell cycle assay and found that the treatment of MoS_2_ NDs under HG/Hypo conditions affected the ratio of each cell cycle phase (G0G1, G2M, and S phases). The result showed that fewer endothelial cells were in the G0-G1 phase (HG/Hypo + PBS = 81.25 ± 4.45% vs. HG/Hypo + MoS_2_ NDs = 67.11 ± 4.87%, *P* = 0.006) and more in the S phase (HG/Hypo + PBS = 12.79 ± 3.25% vs. HG/Hypo + MoS_2_ NDs = 26.04 ± 3.82%, *P* = 0.042) in the MoS_2_ NDs group under HG/Hypo conditions (Fig. 6C-D). Moreover, The CCK-8 assay revealed that the MoS_2_ NDs (50 µg/mL) significantly promoted endothelial cell proliferation under HG/Hypo conditions (Figure S7), which is consistent with the result of EdU staining (Fig. 6E-F). These results indicated the potential of MoS_2_ NDs to promote endothelial proliferation. Meanwhile, Annexin V/ PI flow cytometry revealed that MoS_2_ NDs reduced the number of apoptotic endothelial cells under HG/Hypo conditions (Fig. 6G-H). The negative cell cycle regulators p16 and p21 were also evaluated by western blotting and Immunofluorescence staining. They were found significantly decreased in MoS_2_ NDs under HG/Hypo conditions (p16: HG/Hypo + PBS vs. HG/Hypo + MoS_2_ NDs = 1.50 ± 0.37, *P* = 0.046; p21: HG/Hypo + PBS vs. HG/Hypo + MoS_2_ NDs = 2.66 ± 0.11, *P* < 0.001) (Fig. 6I-J, Figure S28). Additionally, the protein expression levels of apoptosis-related factors, Bcl-2, Bax, and cleaved caspase-3 were assessed to confirm the impact of MoS_2_ NDs on endothelial apoptosis. MoS_2_ NDs were shown to reduce the Bax and the cleaved caspase 3 levels (Bax: HG/Hypo + PBS vs. HG/Hypo + MoS_2_ NDs = 1.59 ± 0.42, *P* = 0.039; cleaved caspase-3: HG/Hypo + PBS vs. HG/Hypo + MoS_2_ NDs = 3.10 ± 1.16, *P* = 0.041) while increasing Bcl-2 expression (HG/Hypo + MoS_2_ NDs vs. HG/Hypo + PBS = 3.45 ± 0.66, *P* = 0.026) under HG/Hypo conditions (Fig. 6K-L). To confirm the apoptotic effect, the JC-1 staining assay was also performed. The early stages of apoptosis are characterized by a decline in mitochondrial membrane potential, which was detected by the transient release of JC-1 from the aggregates to the monomers. The HG/Hypo conditions induced the release of JC-1 monomers, but MoS_2_ NDs aggregated it again (Fig. 6M). All these results comprehensively confirmed that MoS_2_ NDs could reduce the proportion of apoptotic endothelial cells under HG/Hypo conditions. Muscle sections also showed increased Ki67^+^ signaling and reduced signaling of TUNEL in the MoS_2_ NDs group, confirming that MoS_2_ NDs were involved in cell proliferation and apoptosis in vivo(Figure S29-S30).

### MoS_2_ NDs reversed the inhibition of endothelial autophagy induced by HG/Hypo

**Fig. 7:**
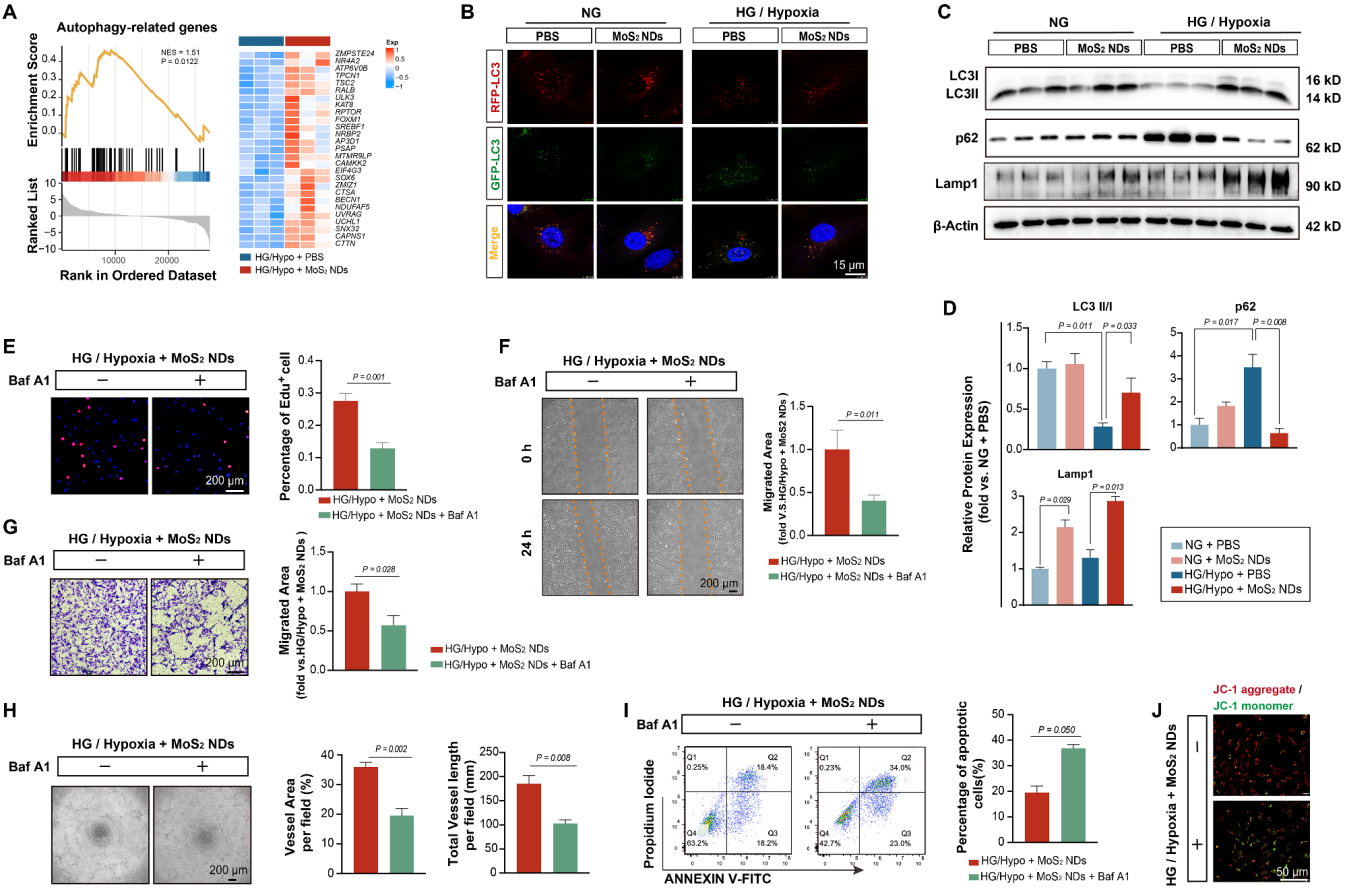
MoS_2_NDs reversed the inhibition of endothelial autophagy induced by HG/Hypo.(**A**) GSEA plot and heatmap of genes related to autophagy. (**B**) Representative images of a single cell expressing RFP-GFP-LC3 following different treatments. (**C**) The expressions of autophagy-related factors: LC3, p62, Lamp1. (**D**) Quantification of protein expression levels with different treatments, *n* = 3, and P values were calculated by two-way ANOVA with Tukey’s post hoc test. (**E**) EdU staining, (**F**) wound healing analysis, (**G**) Transwell analysis, (**H**) tube formation analysis, (**I**) Annexin-V/PI flow cytometric detection, and (**J**) JC-1 staining of HUVECs under HG/Hypo conditions treated with MoS_2_ NDs with or without the autophagy inhibitor, Baf A1, *n* = 3, and P values were calculated by *t*-test. All data are expressed as mean ± SD.

Attenuated oxidative stress in endothelial cells by MoS_2_ NDs is responsible for promoting endothelial cell proliferation, inhibiting apoptosis, and facilitating their neovascularization [19, 29, 40]. Interestingly, we observed an upregulation of autophagy-related genes by MoS_2_ NDs under HG/Hypo conditions (Fig. 5B, Fig. 7A). This could potentially be another factor contributing to the aforementioned benefits. Autophagy plays a protective role in hypoxia-induced angiogenesis [63, 64]. During autophagy, autophagosomes and lysosomes fuse to form autophagolysosomes, which is a prerequisite for autophagy activation. However, studies show that the autophagy activation in endothelial cells is inhibited when exposed to high glucose for prolonged periods owing to the fusion barrier between autophagosomes and lysosomes [65–67]. To assess whether the HG/Hypo conditions inhibited autophagy and whether MoS_2_ NDs restored autophagy, autophagosomes (yellow dots) and autophagolysosomes (red dots) were stained with GFP-RFP-LC3 (Figure S31). The presence of autophagosomes and autophagolysosomes in HUVECs was determined by LSCM. The number of autophagolysosomes per cell decreased in the PBS group under HG/Hypo conditions but increased in the MoS_2_ NDs group, thereby indicating that HG/Hypo conditions inhibited autophagy by interfering with autophagosome-lysosome fusion, whereas MoS_2_ NDs could enable this fusion, thereby restoring autophagy (Fig. 7B, Figure S32-33). We then examined the levels of LC3 (autophagosomes), Lamp1(lysosomes), and p62 to determine changes in autophagic flux [68, 69]. The protein p62 interacts with autophagic substrates and delivers them to autophagosomes for degradation while being degraded itself. LC3 II/I was upregulated (HG/Hypo + MoS_2_ NDs vs. HG/Hypo + PBS = 2.65 ± 1.38, *P* = 0.033), whereas p62 levels (HG/Hypo + PBS vs. HG/Hypo + MoS_2_ NDs = 5.45 ± 1.25, *P* = 0.008) decreased in the MoS_2_ NDs group under HG/Hypo conditions. Lamp1, a marker of lysosome, was upregulated upon MoS_2_ NDs treatment (HG/Hypo + MoS_2_ NDs vs. HG/Hypo + PBS = 2.20 ± 0.31, *P* = 0.013) (Fig. 7C-D). These findings were consistent with p62 and Lamp1 immunofluorescence staining (Figure S34). These results suggested that MoS_2_ NDs activated autophagy under HG/Hypo conditions. Next, we used Bafilomycin A1 (Baf A1), an autophagy inhibitor, to explore whether the angiogenic effect of MoS_2_ NDs was caused by autophagy activation. The EdU staining showed that the involvement of Baf A1 decreased the number of proliferative cells (HG/Hypo + MoS_2_ ND + Baf A1^-^ = 27.60 ± 2.32% vs. HG/Hypo + MoS_2_ NDs + Baf A1^+^ = 12.80 ± 1.81%, *P* = 0.001) (Fig. 7E). The migratory ability of endothelial cells was also blocked by Baf A1, as shown by wound healing and transwell assays (Fig. 7F-G). Moreover, the tube formation assay revealed an impaired tube formation ability with Baf A1 (Fig. 7H). Annexin V/PI flow cytometry revealed an increased number of apoptotic cells with Baf A1 (HG/Hypo + MoS_2_ ND + Baf A1^-^ = 19.50 ± 4.52% vs. HG/Hypo + MoS_2_ NDs + Baf A1^+^ = 36.81 ± 2.49%, *P* = 0.050), as confirmed by JC-1 staining (Fig. 7I-J). The application of another autophagy inhibitor, 3-methyladenine (3-MA) also reversed the angiogenetic effects induced by MoS_2_ NDs (Figure S35). Therefore, we demonstrated that MoS_2_ NDs could improve endothelial cell angiogenesis under HG/Hypo conditions by activating autophagy.

### MoS_2_ NDs activated endothelial autophagy through the cAMP/PKA-NR4A2 signaling pathway

**Fig. 8:**
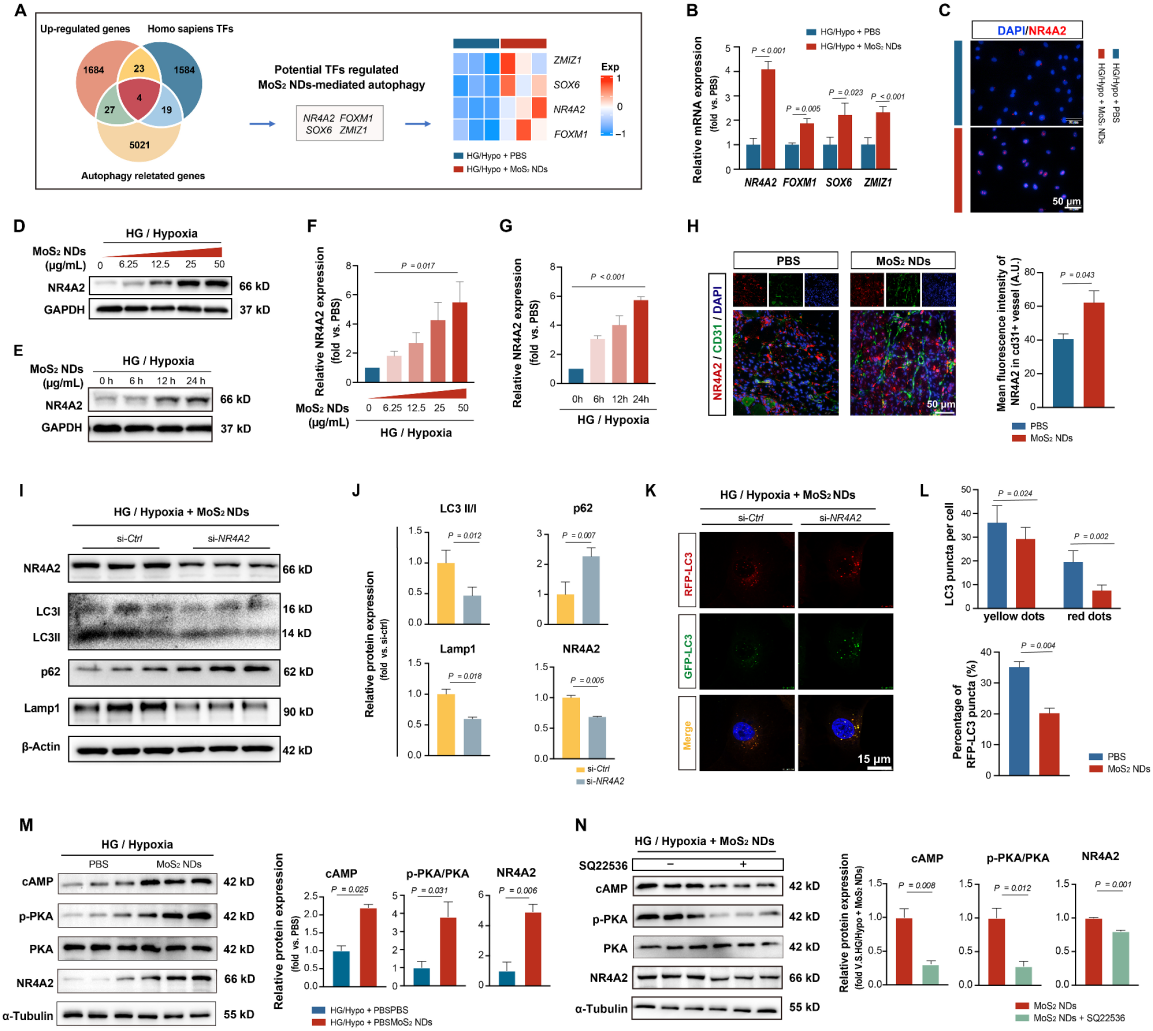
MoS_2_NDs on upregulating autophagy-related transcription factors (TFs) NR4A2 via cAMP/PKA signaling. (**A**) Potential TFs regulating MoS_2_ NDs-mediated autophagy. (**B**) Quantification of the mRNA expression levels of TFs with different treatments, *n* = 3, and P values were calculated by one-way ANOVA with Tukey’s post hoc test. (**C**) Localization of NR4A2 on HUVECs under HG/Hypo conditions with PBS and MoS_2_ NDs, measured by fluorescence microscopy. (**D**) The protein expression levels of NR4A2 with MoS_2_ NDs in a dose-dependent manner and (**E**) time-dependent manner. (**F**) Quantification of NR4A2 protein expression levels with MoS_2_ NDs in a dose-dependent manner and (**G**) in a time-dependent manner, *n* = 3, and P values were calculated by one-way ANOVA. (**H**) Representative images and quantification of the NR4A2 expressions on CD31^+^ vessels with PBS and MoS_2_ ND in vivo. (**I**) The protein expression levels of LC3, p62, Lamp1, and NR4A2 of HUVECs with si-*Ctrl* and si-*NR4A2* under HG/Hypo conditions treated with MoS_2_ NDs. (**J**) Quantification of protein expression levels, *n* = 3, and P values were calculated by *t*-test. (**K**) Representative images of a single cell expressing RFP-GFP-LC3 with si-*Ctrl* and si-*NR4A2*. (**L**) The quantification of autophagosome (yellow dots) and autophagolysosome (red dots) and the proportion of autophagolysosome in all LC3 puncta, *n* = 6, and P values were calculated by one-way ANOVA with Tukey’s post hoc test and *t*-test. (**M**) The related expressions of cAMP, p-PKA, and PKA with PBS and MoS_2_ NDs under hypoxia/high glucose condition, *n* = 3, per group, and P values were calculated by *t*-test. (**N**) The related expressions of cAMP, p-PKA, and PKA under hypoxia/high glucose treated with MoS_2_ NDs with or without the cAMP inhibitor, SQ22536, *n* = 3, per group, and P values were calculated by *t*-test. All data are expressed as mean ± SD.

To explore the mechanisms by which MoS_2_ NDs promoted autophagy under HG/Hypo conditions, we identified four autophagy-related TFs (ZMIZ1, SOX6, NR4A2, FOXM1) [70–74] that were upregulated among the differential genes according to the RNA-sequencing (Fig. 8A). RT-qPCR results showed that MoS_2_ NDs upregulated the mRNA levels of all four genes in a dose- and time-dependent manners (Fig. 8B, Figure S36-S37), with NR4A2 exhibiting the highest increases. Immunofluorescence staining revealed that NR4A2 was highly expressed in the nuclei of cells in the MoS_2_ NDs group (Fig. 8C). The protein expression of NR4A2 also increased in a dose- and time-dependent manner (Fig. 8D-G). Immunofluorescence staining of mice muscle sections after HLI showed higher NR4A2^+^ signaling in the CD31^+^ area in the MoS_2_ NDs group (Fig. 8H). All the above data demonstrated that MoS_2_ NDs significantly upregulated NR4A2 in vitro and in vivo. To explore whether NR4A2 mediates MoS_2_ NDs-induced autophagy, we constructed a small interfering RNA of NR4A2 (si-*NR4A2*) and confirmed its ability to knockdown the protein expression level of NR4A2 in HUVECs (Figure S38). We then evaluated the impact of knocking down NR4A2 on autophagy. When exposed to HG/Hypo conditions with MoS_2_ NDs, there was a significant decrease in autophagy in the si-*NR4A2* group, which was demonstrated by a decrease in the LC3 II/I ratio, an increase in the p62 level, and a decrease in the Lamp1 level (Fig. 8I-J). The RFP-GFP-LC3 assay also showed fewer red dots (autophagolysosome) in the si-*NR4A2* group, indicating the inhibition of autophagy (Fig. 8K-L, Figure S39). These data confirmed the involvement of NR4A2 in autophagy activation.

Previous studies reported that MoS_2_ could upregulate cAMP levels in macrophages [75]. Moreover, cAMP levels triggered cAMP/PKA signaling [76], which could activate NR4A2 upregulation [77, 78]. However, cAMP/PKA signaling is significantly impaired in diabetes [79]. Accordingly, we hypothesized that MoS_2_ NDs enter endothelial cells, increase cAMP levels, and activate cAMP/PKA signaling, thus leading to NR4A2 upregulation under high glucose and hypoxic conditions. Our experiments thus demonstrated that MoS_2_ NDs upregulated cAMP level (HG/Hypo + MoS_2_ NDs vs. HG/Hypo + PBS = 2.20 ± 0.15, *P* = 0.025), which was accompanied by an increase in phosphorylated PKA (HG/Hypo + MoS_2_ NDs vs. HG/Hypo + PBS = 3.81 ± 1.43, *P* = 0.031) and an increase in NR4A2 (HG/Hypo + MoS_2_ NDs vs. HG/Hypo + PBS = 4.92 ± 0.86, *P* = 0.006) (Fig. 8M). However, the upregulated expression of NR4A2 by MoS_2_ NDs was blocked by the cAMP inhibitor, SQ22536, thereby demonstrating that the cAMP/PKA signaling pathway is responsible for the upregulation of NR4A2 (Fig. 8N) [75–79].

### Nuclear receptor NR4A2 regulated autophagy by transcriptionally activating autophagy-associated genes ATG4B, ATG9B, and ATG13

**Fig. 9:**
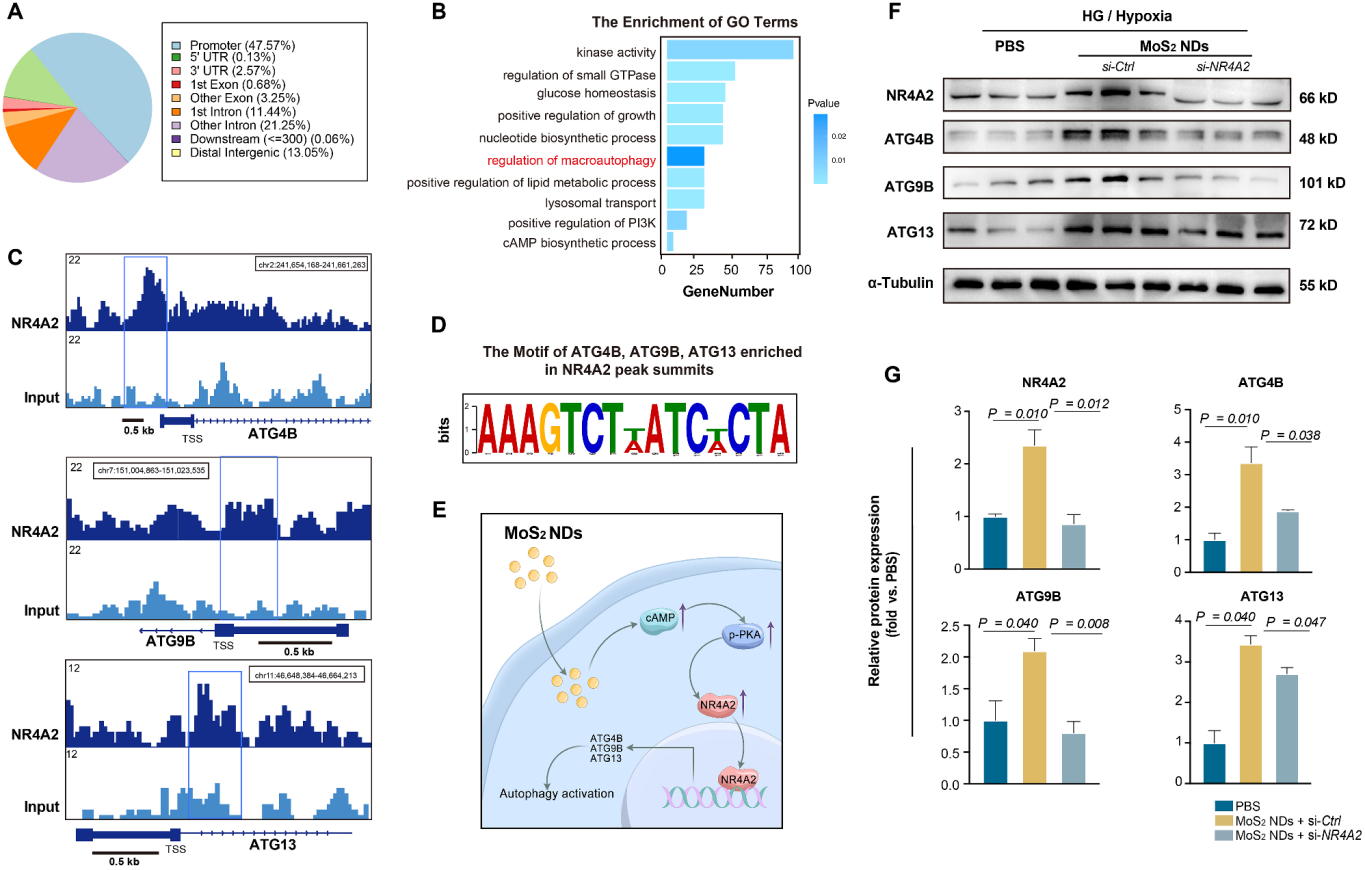
Nuclear receptor NR4A2 activated autophagy by up-regulating autophagy-associated genes ATG4B, ATG9B, and ATG13.(**A**) The positions of NR4A2-binding peaks on a genome-wide scale in ChIP-seq results. (**B**) The GO enrichment analysis for NR4A2 occupancy. (**C**) Gene tracks of NR4A2 enrichment by ChIP-seq analysis at core promoter regions of ATG4B, ATG9B, and ATG13. (**D**) The most significant NR4A2-binding motif of ATG4B, ATG9B, and ATG13 identified by ChIP-seq results. (**E**) Schematic illustration of MoS_2_ NDs-mediated autophagy activation in HUVECs. (**F**) The related expressions of ATG4B, ATG9B, and ATG13 with PBS and MoS_2_ NDs with or without NR4A2 knockdown. (**G**) Quantification of protein expression levels with different treatments, *n* = 3, per group, and P values were calculated by one-way ANOVA with Tukey’s post hoc test. All data are expressed as mean ± SD.

Despite substantial evidence from previous studies reporting the involvement of NR4A2 in the regulation of autophagy [72, 73], few studies have investigated the definitive mechanism by which NR4A2 regulates autophagy. As a transcription factor, NR4A2 binds to DNA fragments. Therefore, we analyzed the ChIP-seq of the NR4A2 from the Gene Expression Omnibus database. The ChIP-seq analysis identified 4799 binding peaks, most of which (47.57%) were located in the promoter regions (Fig. 9A). Genes matched by the binding peaks in the promoter regions were enriched in the autophagy-related pathway (Fig. 9B). Moreover, we were surprised to find that NR4A2 bound to the promoter regions of ATG4B, ATG9B, and ATG13, which played roles in directly regulating autophagosome formation and autophagosome-lysosome fusion (Fig. 9C) [80–82]. The most significant NR4A2-binding motif in ATG4B, ATG9B, and ATG13 was also identified by ChIP-seq results (Fig. 9D). Figure 9E comprehensively illustrates how MoS_2_ NDs upregulate NR4A2 after entering the endothelial cells and how NR4A2 activates autophagy. The western blotting results in Fig. 9F demonstrated that MoS_2_ NDs could upregulate the protein expression of ATG4B, ATG9B, and ATG13 along with NR4A2 under HG/Hypo conditions. However, MoS_2_ NDs did not upregulate the expression of the three factors when the level of NR4A2 was reduced by small interfering RNA (Fig. 9F-G), indicating that the increase in levels of ATG4B, ATG9B, and ATG13 can be attributed to the upregulation of NR4A2. In summary, MoS_2_ NDs increase NR4A2 expression *via* cAMP/PKA signaling and increase NR4A2 binding to the promoter regions of ATG4B, ATG9B, and ATG13, thereby increasing the expression of these factors and activating autophagy. Therefore, based on the aforementioned results, we have elucidated a molecular mechanism by which MoS_2_ nanomaterials promote endothelial cell autophagy.

Collectively, we demonstrated the potential effects of MoS_2_ NDs on enhancing collateral formation in T2DM patients. MoS_2_ NDs have the advantages of rapid metabolism, low body retention, and high biosafety. Through its antioxidant-like enzyme activity and activation of autophagy, it alleviates endothelial cell dysfunction and thus promotes endothelial angiogenesis in diabetes.

Existing approaches for promoting angiogenesis include pro-angiogenic factor therapy and cell therapy [11, 16]. Pro-angiogenic factor therapy aims to activate signaling pathways related to angiogenesis, while cell therapy involves the supplementation of cells with angiogenic capabilities to promote angiogenesis. However, these therapies are associated with high costs, limited availability, and susceptibility to degradation [11, 12]. Additionally, they have inherent deficiencies in diabetes treatment due to impaired angiogenic pathways and the detrimental effects of a high glucose environment on transplanted cells [14, 15, 17]. Conversely, MoS_2_ NDs offer a convenient synthesis method, use inexpensive raw materials (Fig. 1), and have demonstrated excellent stability (Figure S3, S6, S14). Moreover, these nanodots specifically treat diabetes-induced oxidative stress (Fig. 3G-H) and the inhibition of autophagy (Fig. 7A-C), thereby restoring endothelial cell function to promote angiogenesis. These advantages highlight the immense potential of MoS_2_ NDs compared to existing therapies.

Although previous studies have demonstrated the wide-ranging applications of MoS_2_-based nanomaterials in the field of biomedicine [83–85], our research has made progress specifically in the following two aspects:

First, we explored the potential of MoS_2_ to promote angiogenesis by activating autophagy and uncovered its underlying molecular mechanisms. Firstly, we applied autophagy inhibitors to demonstrate that MoS_2_ NDs promote angiogenesis through the activation of autophagy (Fig. 7E-J). Furthermore, RNA sequencing and molecular biology techniques identified NR4A2 as a potential transcription factor mediating autophagy activated by MoS_2_ NDs (Fig. 8A-L). Experimental validation revealed that NR4A2 is upregulated by MoS_2_ NDs through the activation of the cAMP/PKA signaling pathway (Fig. 8M-N). ChIP-seq analysis of NR4A2 demonstrated its binding to the promoters of ATG4B, ATG9B, and ATG13, promoting their expression and activation of autophagy (Fig. 9). Thus, our study revealed the molecular mechanism of MoS2 NDs activating autophagy through cAMP/PKA-NR4A2.

Second, we propose a specific therapeutic strategy for impaired neovascularization in diabetes resulting from ROS overload and autophagy Inhibition. Two key factors are closely associated with impaired angiogenesis in diabetes: (1) excessive levels of ROS and (2) impaired autophagy [18, 19]. By leveraging the dual therapeutic properties of MoS_2_ NDs (antioxidant and autophagy activation), we aim to mitigate the detrimental effects of ROS and enhance autophagy, thus promoting angiogenesis with diabetes. This treatment strategy and its demonstrated effectiveness in the article also hold promise for the treatment of a range of complications caused by diabetes, such as diabetic nephropathy, diabetic neuropathy, and diabetic vascular disorders, which are associated with high levels of oxidative stress and autophagic damage.

Nevertheless, this study has some limitations. First, the ultra-small size of MoS_2_ NDs facilitates their clearance and metabolism, which improves biosafety but shortens the circulation time of MoS_2_ NDs, thereby limiting their effect. Therefore, our future study will employ an engineering strategy to coat biomimetic cell membranes with MoS_2_ NDs to prolong their circulation time [86]. Second, the size, thickness, and surface modification may affect the activity of the MoS_2_ NDs. This study highlights the prospects of applying the MoS_2_ NDs for treating T2DM with atherosclerotic diseases and only applies one type of reported MoS_2_ NDs to confirm their autophagy activation and ROS scavenging properties. Therefore, further studies should focus on activity regulation by using a broader assessment of the different physiochemical properties of MoS_2_ NDs.

## Conclusions

In summary, the ultra-small MoS_2_ NDs were considered a therapeutic nano-agent for impaired collateral growth in T2DM by combining nanocatalysis therapy with autophagy amplification. Moreover, A specific mechanism (cAMP-NR4A2) has been uncovered to substantiate the effect of MoS_2_ NDs in promoting autophagy. The clinical potential and applicability of MoS_2_ NDs are evident in two distinct aspects. Firstly, the fabricated MoS_2_ NDs exhibited remarkable renal clearance efficiency along with superior biosafety. Should future research affirm their biosecurity in larger animals and potentially in human subjects, MoS_2_ NDs could unlock significant translational opportunities. Secondly, the therapeutic efficacy of MoS_2_ NDs, as evidenced in our study, extends beyond the realm of diabetes complications. It opens avenues for promising treatments in a spectrum of disorders characterized by deficits in autophagy and elevated oxidative stress. This broadens the scope of MoS_2_ NDs, positioning these ultra-small bioactive nanodots as a kind of versatile candidate in the landscape of therapeutic interventions.

### Electronic supplementary material

Below is the link to the electronic supplementary material.

Supplementary Material 1

## Data Availability

No datasets were generated or analysed during the current study.

## Abbreviations

3-MA: 3-methyladenine
AGEs: advanced glycation end products
ASCVDs: atherosclerotic cardiovascular diseases
AUC: area under curve
Baf A1: bafilomycin A1
CAT: catalase
DAPI: 4′,6-diamidino-2-phenylindole
CCK-8: cell counting kit-8
DAF-FM-DA: 3-amino,4-aminomethyl-2’:7’-fluorescein diacetate
ChIP-seq: chromatin immunoprecipitation sequencing
DCFH-DA: dichlorodihydrofluorescein diacetate
DHE: dihydroethidium
DI water: deionized water
DMEM: dulbecco’s modified eagle medium
DMPO: 5,5-dimethyl-1-pyrroline N-oxide
ECGS: endothelial cell growth supplement
EDTA: ethylenediaminetetraacetic acid
EdU: 5-ethynyl-2- deoxyuridine
EPR: enhanced permeability and retention
ESR: electron spin resonance
FBS: fetal bovine serum
FDR: false discovery ratio
FGF: fibroblast growth factor
FITC: fluorescein isothiocyanate
FT-IR: fourier-transform infrared spectroscopy
GO: Gene Ontology
GSEA: Gene Set Enrichment Analysis
H&E: hematoxylin-eosin
HFD: high-fat diet
HG/Hypo: high glucose and hypoxia
HLI: hind limb ischemia
HUVECs: human umbilical vein endothelial cells
•OH: hydroxyl radical
•O_2_^-^: superoxide anion
PDGF: platelet-derived growth factor
ICP-OES: inductively coupled plasma-optical emission spectrometer
%ID/g: percentages of the tissue-injected dose per gram
JC-1: 5,5’,6,6’-tetrachloro-1,1’,3,3’-tetraethylbenzimidazolylcarbocyanine iodide
KEGG: Kyoto encyclopedia of genes and genomes
LSCM: laser scanning confocal microscopy
MDA: malondialdehyde
MoS_2_: molybdenum disulfide
MoS_2_ NDs: molybdenum disulfide nanodots
NG: normal glucose; [(NH_4_)_2_MoS_4_]:ammonium tetrathiomolybdate
NO: itric oxide
PBS: phosphate buffer saline
PFA: paraformaldehyde
PI: propidium iodide
PVP: polyvinyl pyrrolidone
SOD: superoxide dismutase
STZ: streptozotocin
RT-qPCR: quantitative reverse transcription polymerase chain reaction
T2DM: type 2 diabetes mellitus
TEM: transmission electron microscopy
TUNEL: terminal deoxynucleotidyl transferase dUTP nick end labeling
VEGF: vascular endothelial growth factor
XPS: X-ray photoelectron spectroscopy
XRD: X-ray diffraction
