# Supplementary material for: Promoting collateral formation in type 2 diabetes mellitus using ultra-small nanodots with autophagy activation and ROS scavenging
Source: J Nanobiotechnology. 2024 Mar 1;22:85. doi: 10.1186/s12951-024-02357-z (PMC10908163; doi:10.1186/s12951-024-02357-z)
Supplement: Supplementary file 1 — Supplementary Material 1 [file 12951_2024_2357_MOESM1_ESM.docx]

**Supplementary Information**

**Promoting Collateral Formation in Type 2 Diabetes Mellitus Using Ultra-Small Nanodots with Autophagy Activation and ROS Scavenging**

Yixuan Wang, ^1,2 #^ Feifei Li, ^1,2 #^ Linshuang Mao, ^1,2^ Yu Liu, ^3^ Shuai Chen, ^1,2^ Jingmeng Liu, ^1,2^ Ke Huang, ^1,2^ Qiujing Chen, ^1,2^ Jianrong Wu, ^4^ Lin Lu, ^1,2^ Yuanyi Zheng, ^4^ Weifeng Shen, ^1,2^ Tao Ying, ^4 *^ Yang Dai, ^1,2 *^ and Ying Shen^1,2 *^

^1^Department of Cardiovascular Medicine, Rui Jin Hospital, Shanghai Jiao Tong University School of Medicine, Shanghai 200025, China

^2^Shanghai Clinical Research Center for Interventional Medicine, Shanghai 200025, China

^3^Beijing Advanced Innovation Center for Big Data-Based Precision Medicine, School of Medicine and Engineering, Beihang University, Beijing 100191, China

^4^Department of Ultrasound in Medicine, Shanghai Sixth People's Hospital Affiliated to Shanghai Jiao Tong University School of Medicine, Shanghai 200233, China

^#^ These authors contributed equally to this study.

^*^Corresponding authors. Email: yingtaomail@yeah.net, yutongwushe@163.com, rjshenying8@163.com

**Supplementary Figures**


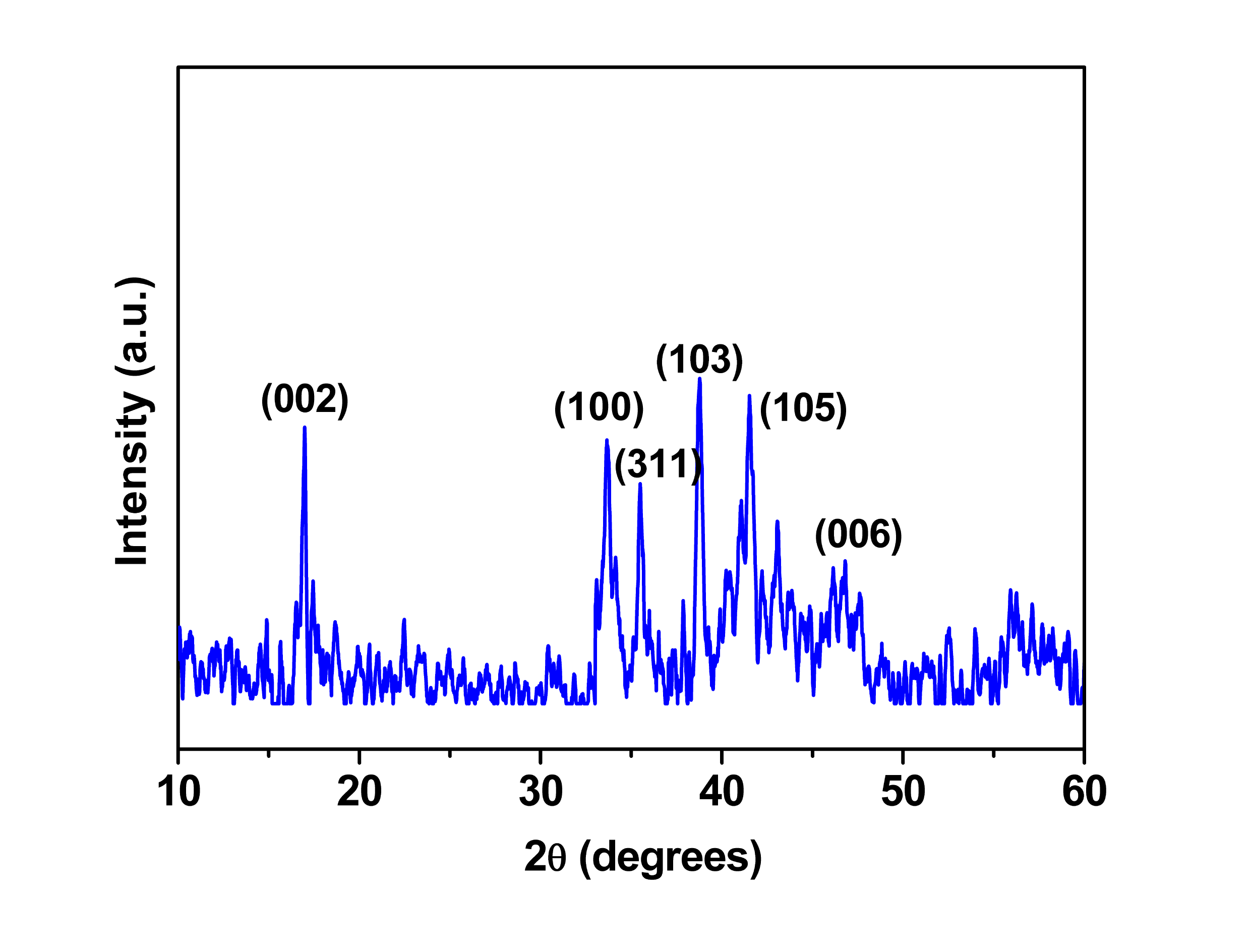


**Figure S1**. The XRD pattern of MoS_2_ NDs demonstrates the good crystallinity of MoS_2_ NDs.

**
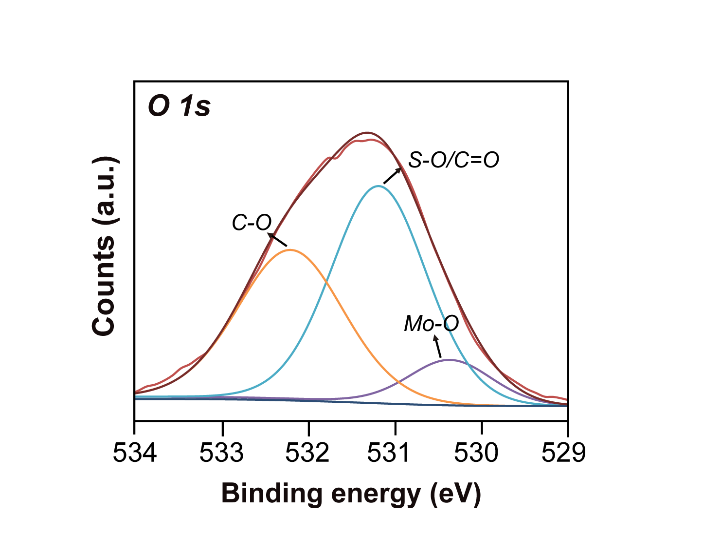
**

**Figure S2.** XPS data showing the spectrum of O 1s.


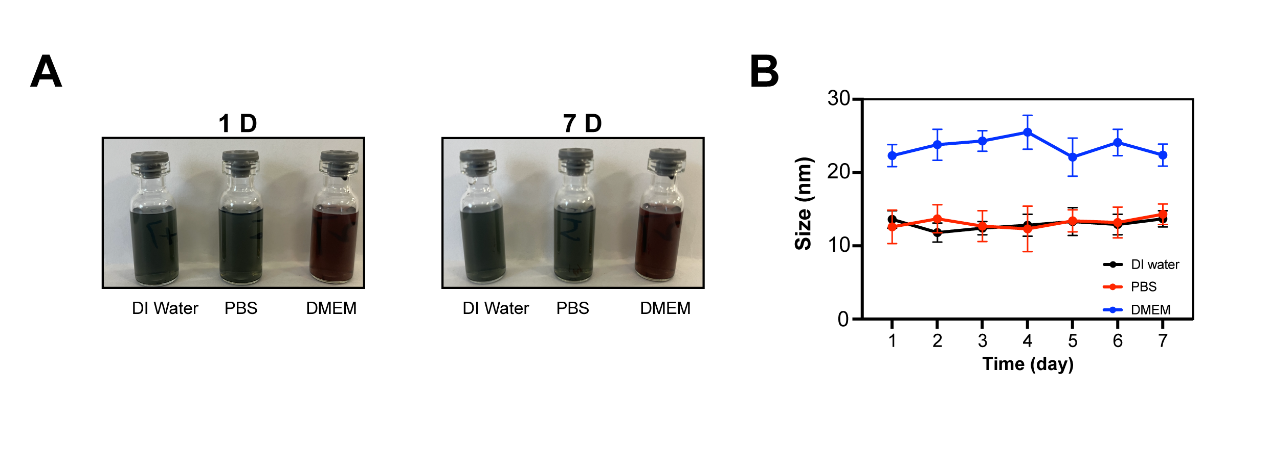


**Figure S3. Stability of MoS_2_ NDs in different media for 7 days.** (A) MoS_2_ NDs dissolved in DI water, PBS, and DMEM on days 1 and 7. (B) The size of MoS_2_ NDs for different days, n = 3, per group.


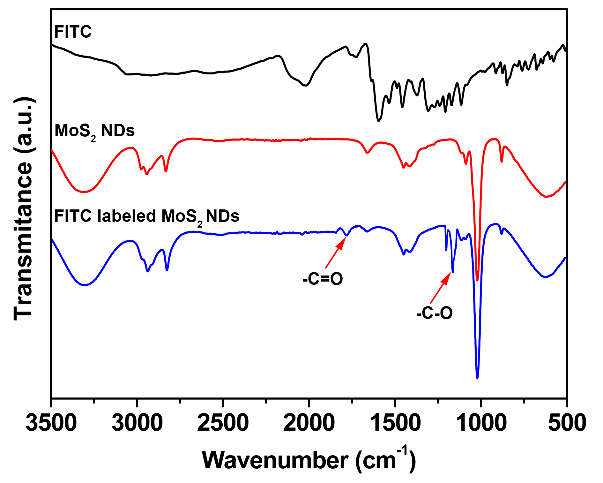


**Figure S4.** The FT-IR spectra of FITC, MoS_2_ NDs, and FITC-MoS_2_ NDs.


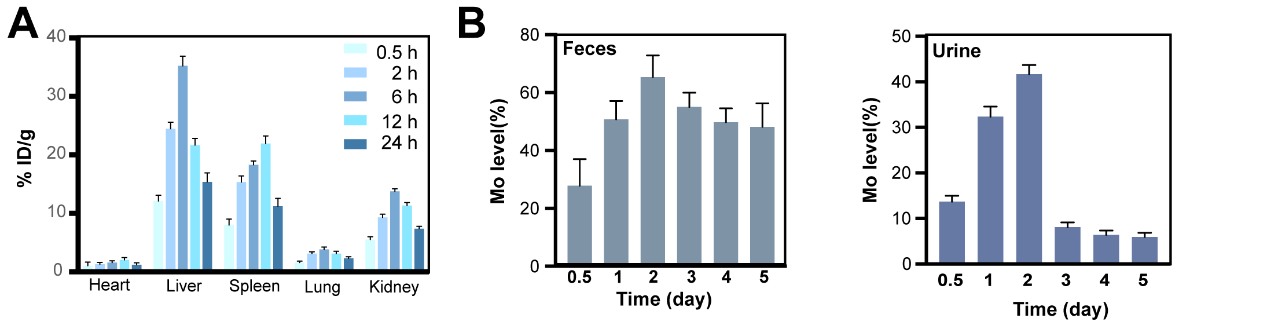


**Figure S5.** ***In vivo* biodistribution and metabolism performance of MoS_2_ NDs.** (A) Biodistribution of MoS_2_ in the organs at different time points, n = 3 per group. (B) Mo level in feces and urine at various time intervals, n = 3 per group. The data are presented as the mean ± SD.


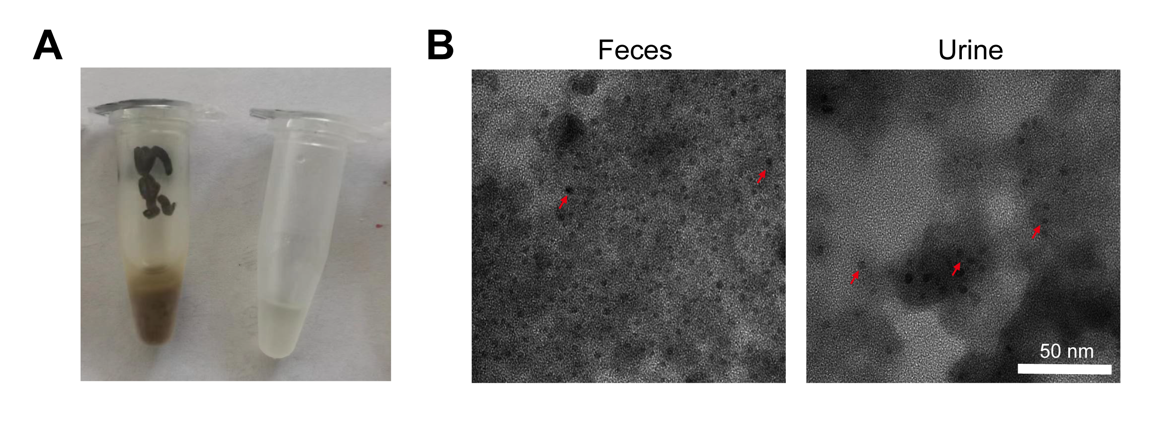


**Figure S6. The MoS_2_ NDs in feces and urine.** (A) Droplet samples of feces and urine for TEM. (B) TEM images of MoS_2_ NDs dispersed in Feces and Urine. Urine and feces were collected from 24 to 48 hours after the injection of MoS_2_ NDs.


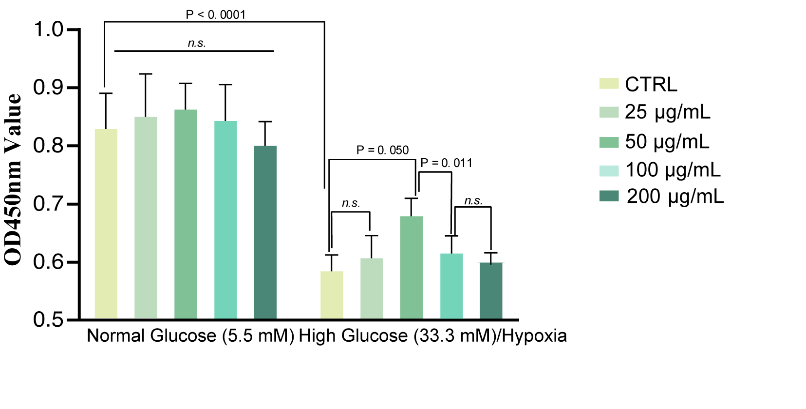


**Figure S7. CCK-8 assay of MoS_2_ NDs**. The CCK-8 assay of MoS_2_ NDs on HUVECs under normal glucose (NG) and high glucose/hypoxia (HG/Hypo) conditions with different concentrations, n = 6 per group. The data are presented as the mean ± SD, and P values were calculated by two-way ANOVA with Tukey’s post hoc test.


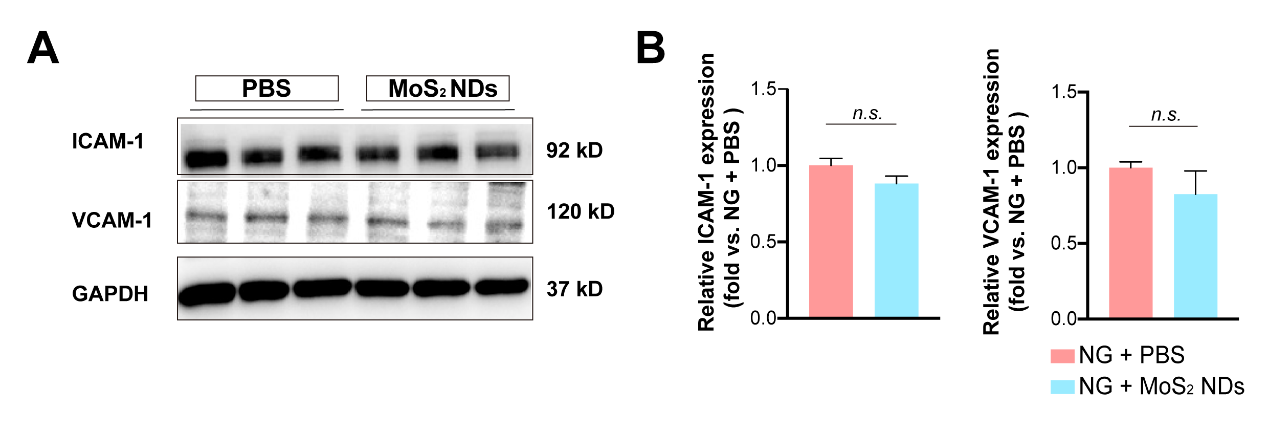


**Figure S8. Expression of inflammation-related protein.** (A) ICAM-1 and VCAM-1 expression in HUVEC in the PBS group and MoS_2_ NDs group. (B) Comparison of the protein expression levels of ICAM-1 and VCAM-1, n = 3 per group. The data are presented as the mean ± SD, and P values were calculated by *t*-test.


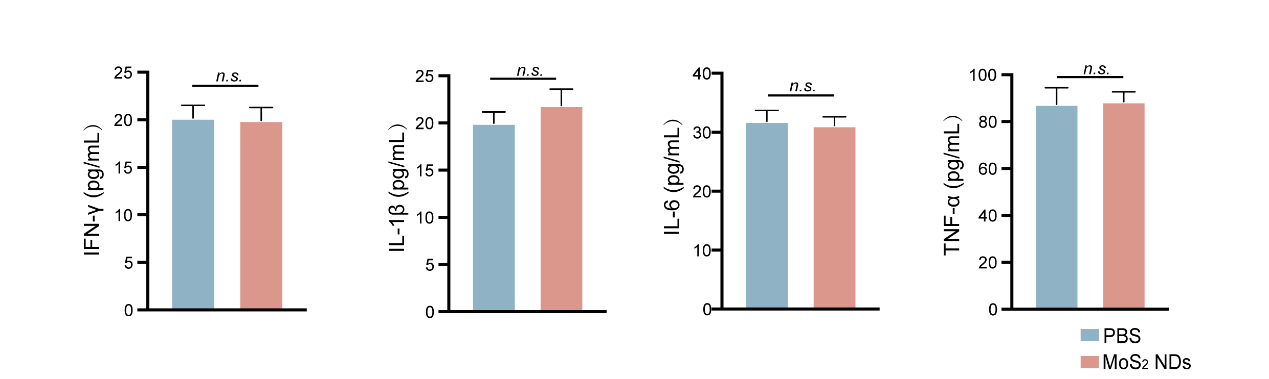
**Figure S9.** The IFN-γ, IL-1β, IL-6, and TNF-α levels in mice serum after being treated with PBS and MoS_2_ NDs. n = 18 per group. The data are presented as the mean ± SD, and P values were calculated by *t*-test.


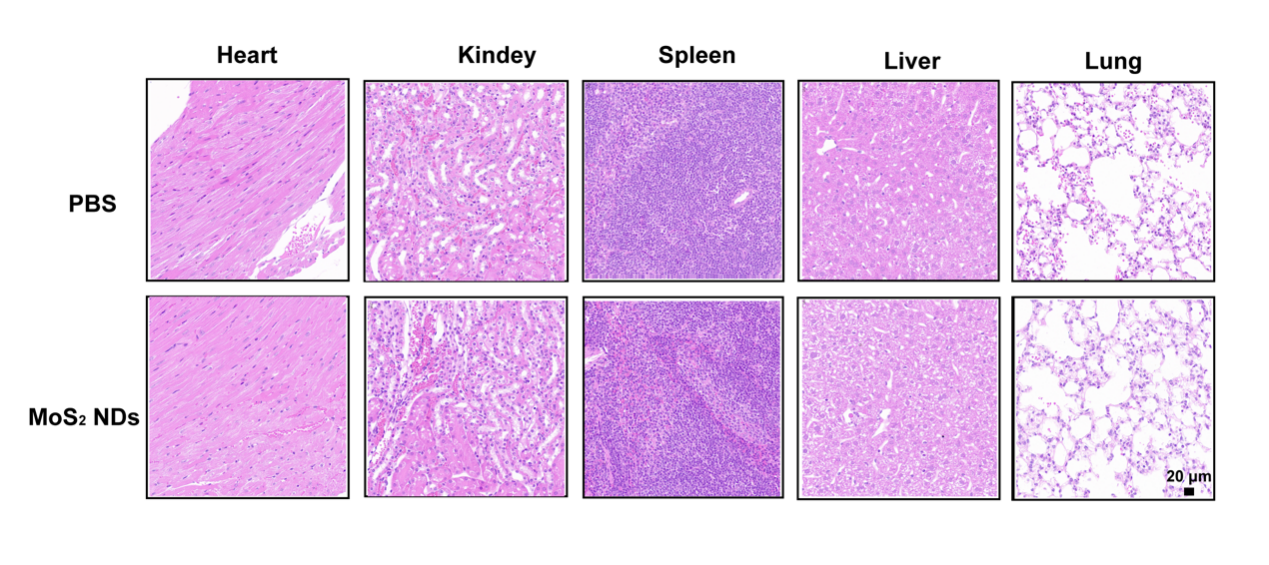


**Figure S10.** *In vivo* biocompatibility assessment of MoS_2_ NDs by major organs H&E sections.


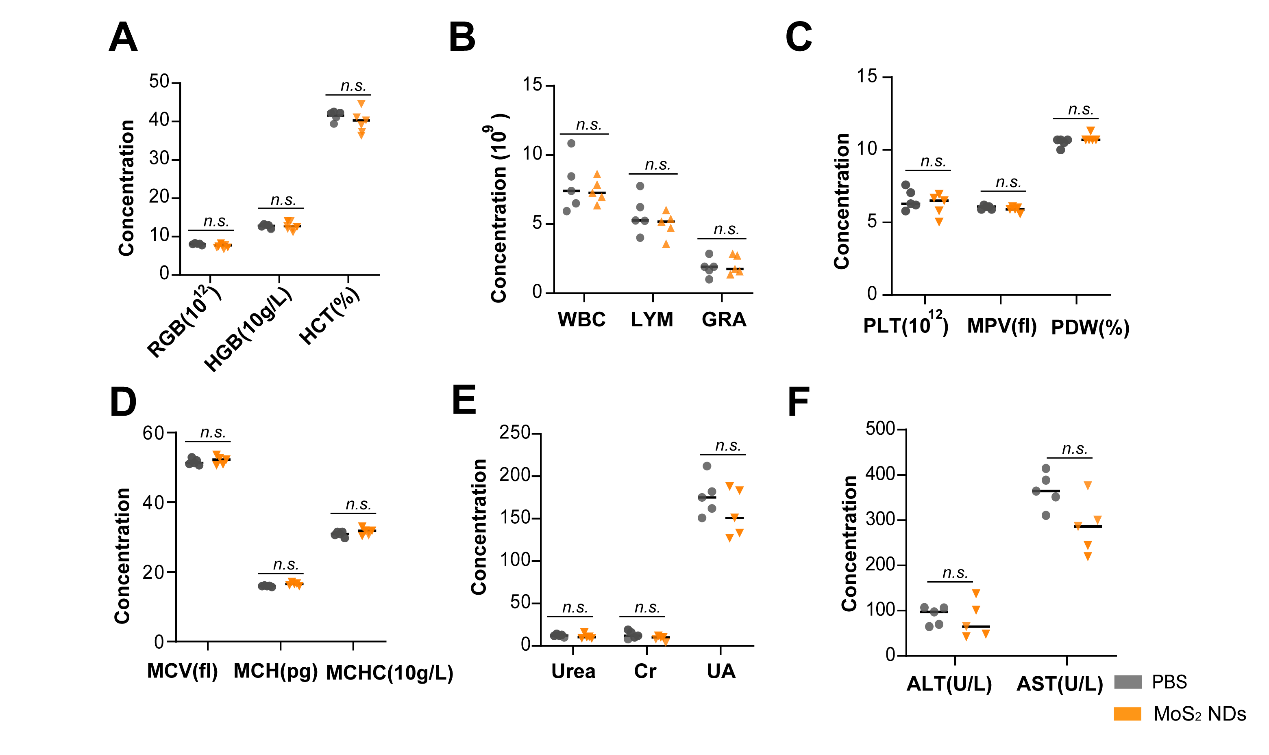


**Figure S11.** ***In vivo* biocompatibility of MoS_2_ NDs.** (A-D) Complete blood count indicators of mice with PBS and MoS_2_ NDs: red blood cell (RBC), hemoglobin (HGB), and red blood cell-specific volume (HCT); white blood cell (WBC), lymphocyte (LYM), and granulocyte (GRA); mean corpuscular volume (MCV), mean corpuscular hemoglobin (MCH), mean corpuscular hemoglobin concentration (MCHC). (E) Renal function index: urea, creatinine (Cr), uric acid (UA). (F) Liver function index: alanine transaminase (ALT), aspartate transaminase (AST). The data are presented as the mean ± SD, and P values were calculated by one-way ANOVA with Tukey’s post hoc test.


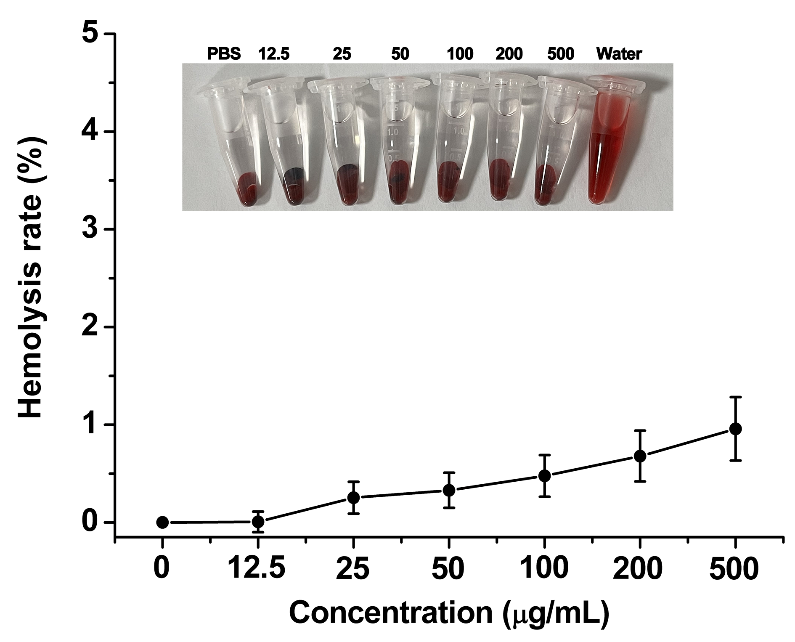


**Figure S12**. Hemolysis rate of MoS_2_ NDs at different concentrations. Inset: digital photograph of hemolysis test.

***
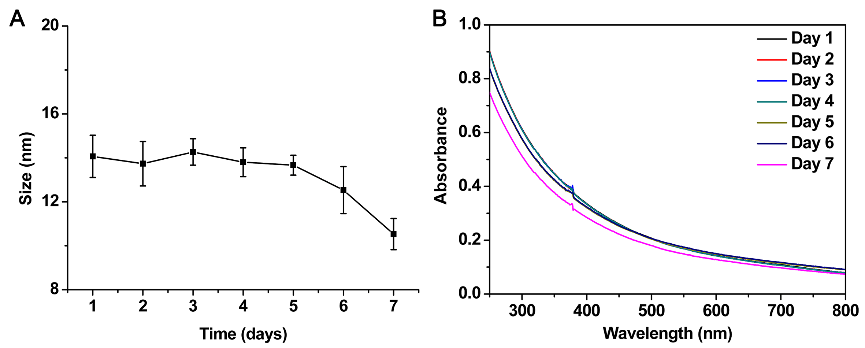
***

**Figure S13.** The (A) hydrodynamic size and (B) UV–vis absorption spectra of MoS_2_ NDs dispersed in acid and oxidizing environment (pH 5.0, 200 mM H_2_O_2_) for 7 days

**
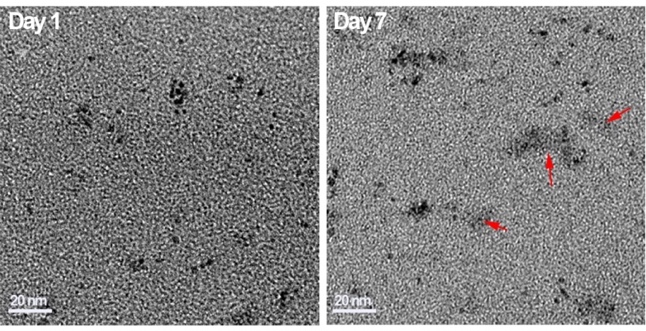
**

**Figure S14.** TEM images of MoS_2_ NDs dispersed in acid and oxidizing environment (pH 5.0, 200 mM H_2_O_2_) for 1 and 7 days.


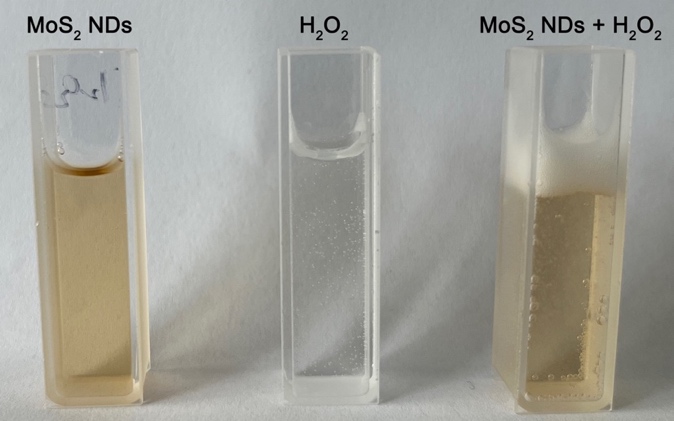


**Figure S15.** The O_2_ production rate of MoS_2_ NDs with different concentrations, n = 3.


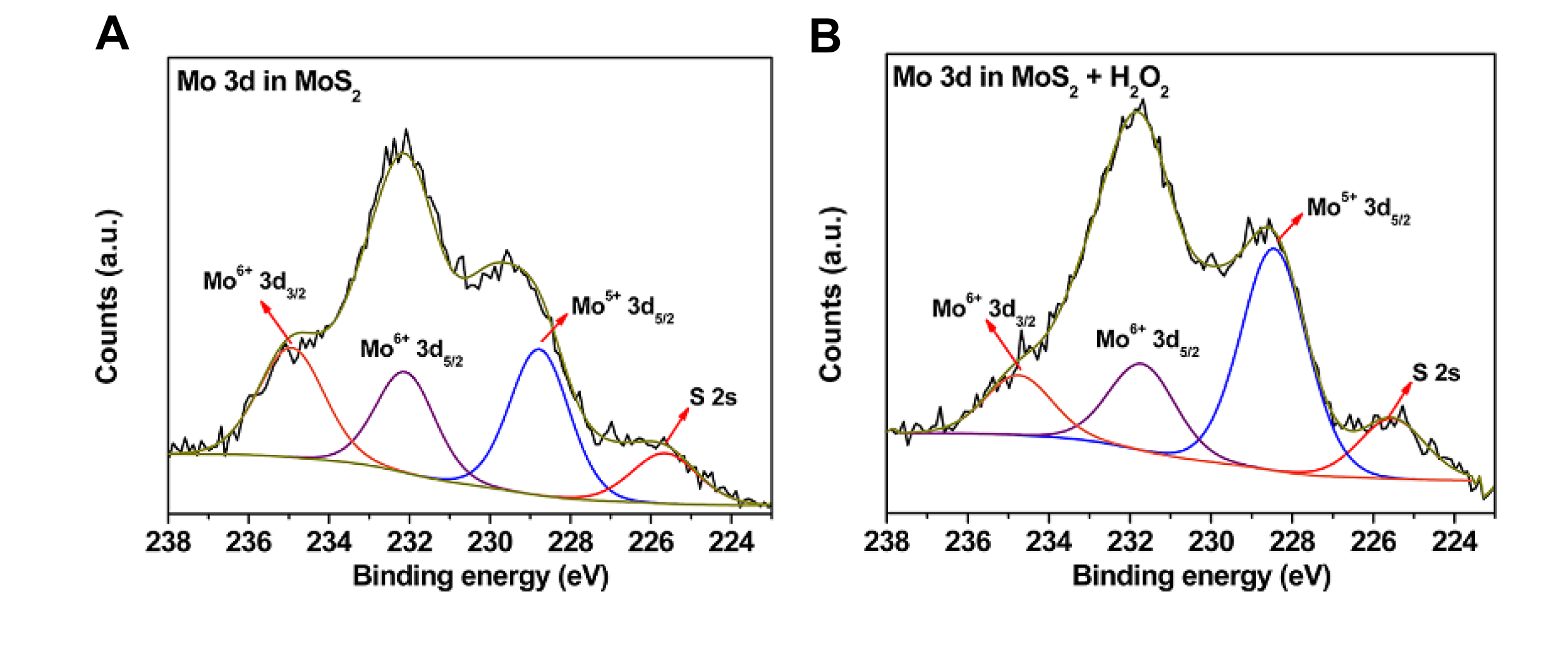


**Figure S16.** Mo3d XPS spectra of MoS_2_ NDs (a) before and (b) after the addition of H_2_O_2_ (50 mM).


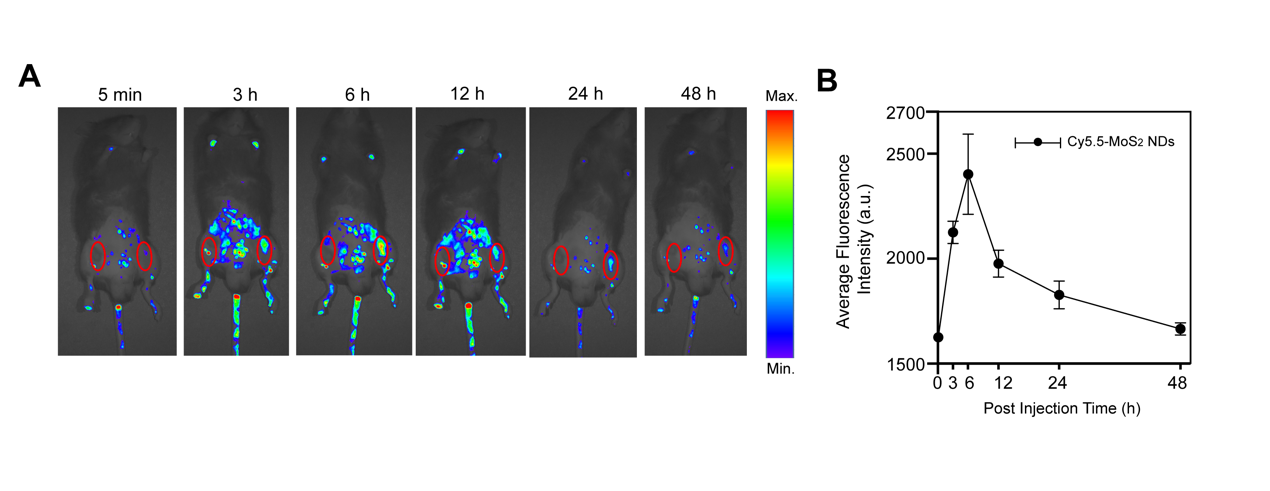


**Figure S17.** (A) *In vivo* fluorescence image of mice after hindlimb ischemia at different time points after intravenous injection of Cy5.5-MoS_2_ NDs, and (B) quantitative analysis of fluorescence intensity per unit in the ischemic area.


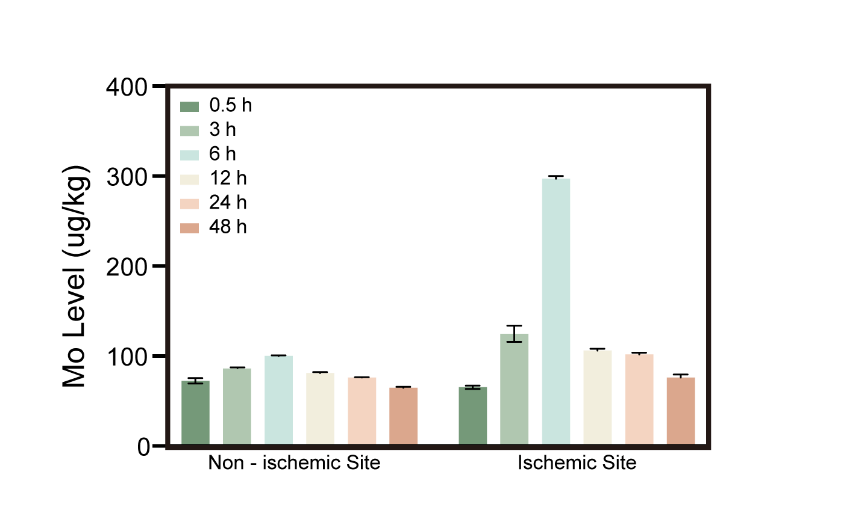


**Figure S18.** Biodistribution of MoS_2_ NDs in the non-ischemic muscle and ischemic muscle at different time points, n = 3 per group.


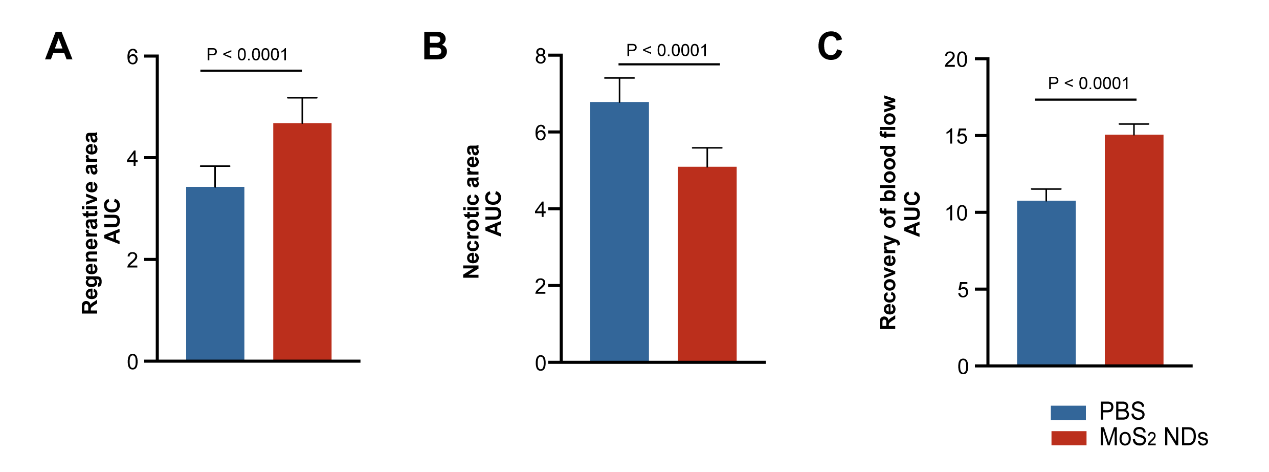


**Figure S19.** Areas under the curve (AUC) of (A) regenerative area, (B) necrotic area, and (C) recovery of blood flow of diabetic mice after hindlimb ischemia with PBS or MoS_2_ NDs, n = 6, per group. The data are presented as the mean ± SD, and P values were calculated by *t*-test.


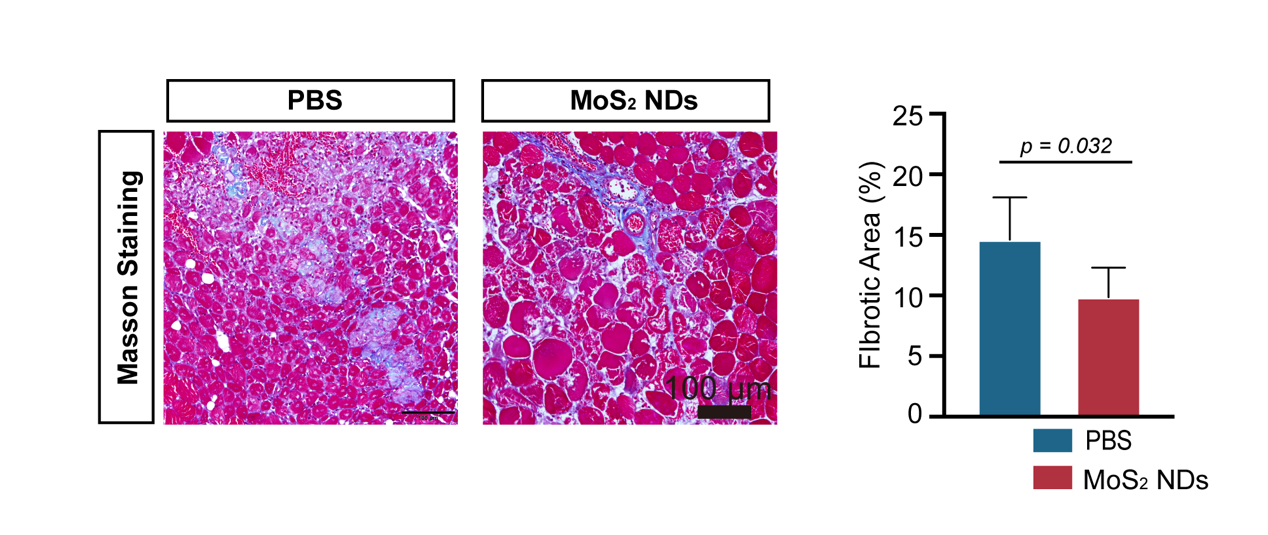


**Figure S20**. Masson staining of muscle section on day 21 after hindlimb ischemia, and quantitative analysis of the fibrotic area of the muscle section, n = 9, P values were calculated by *t*-test.


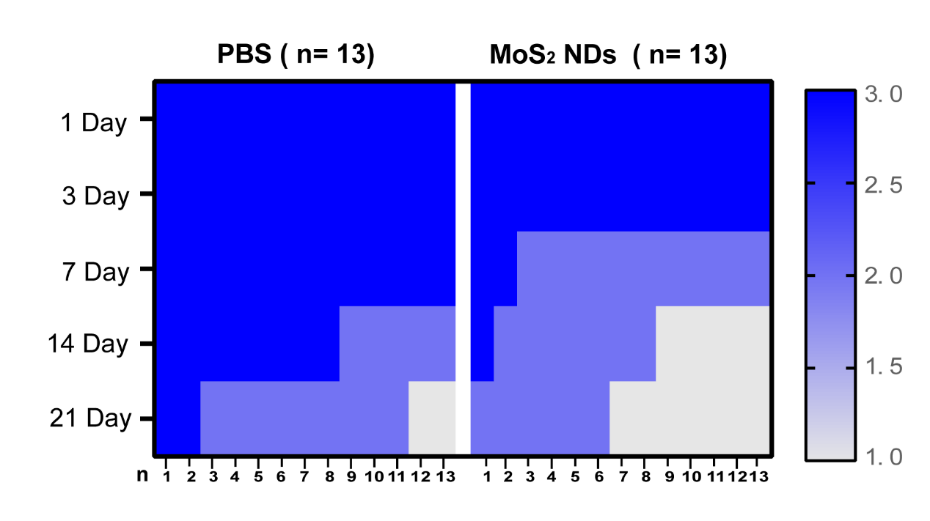


**Figure S21.** Ischemic damage score of diabetic mice after hindlimb ischemia with PBS and MoS_2_ NDs.


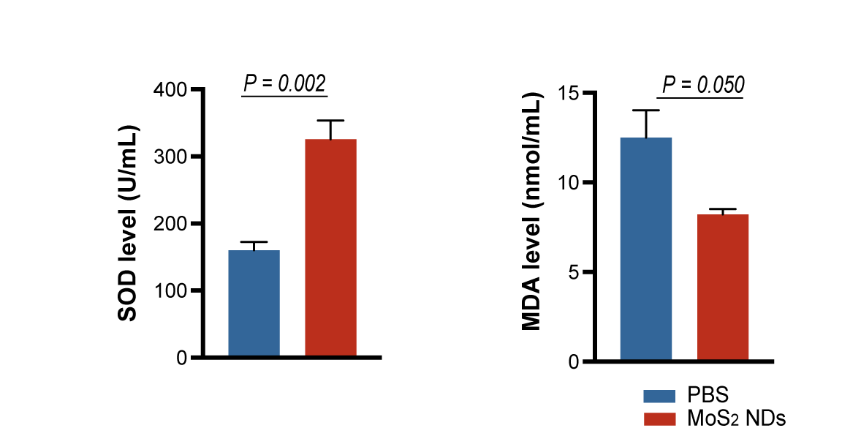


**Figure S22.** Serum of SOD and MDA levels of diabetic mice with PBS or with MoS_2_ NDs on day 7, n = 6 per group, the data are presented as the mean ± SD, and P values were calculated by *t*-test.


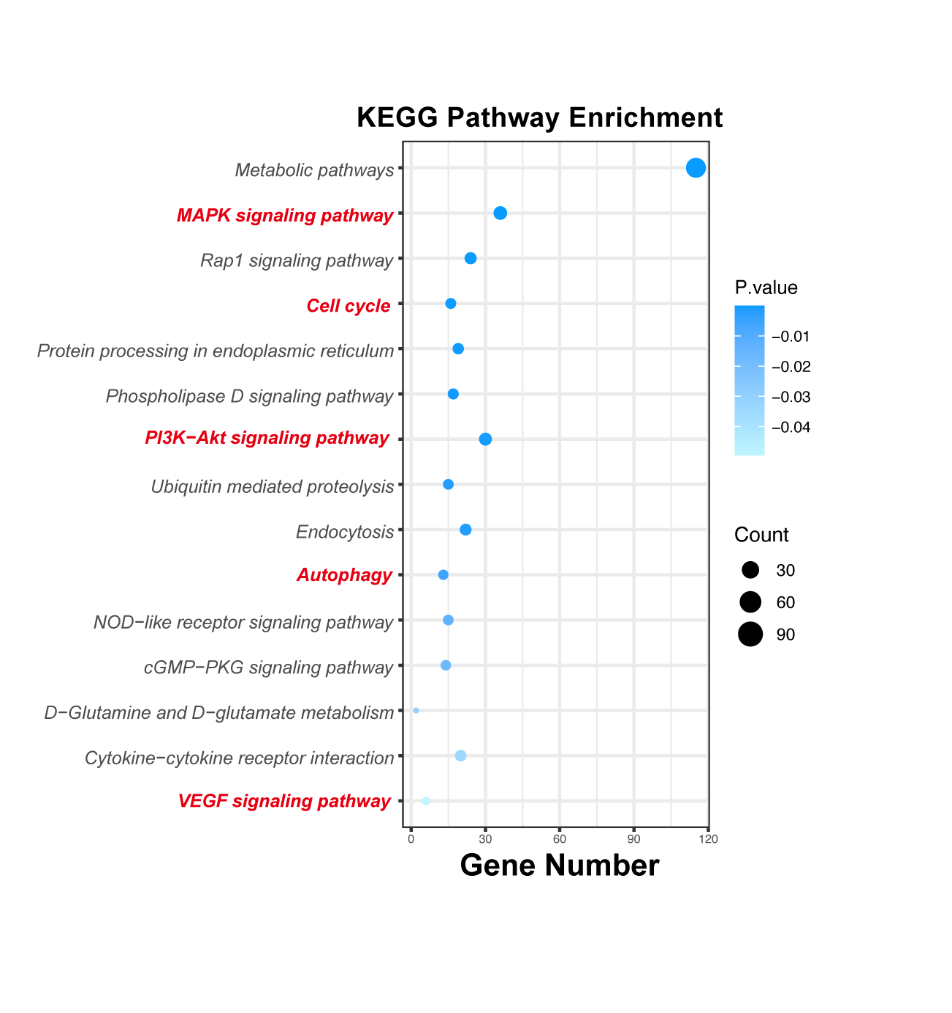


**Figure S23.** Differential expression genes of HUVECs between the PBS group and MoS_2_ group enriched for pathways using KEGG enrichment analysis.


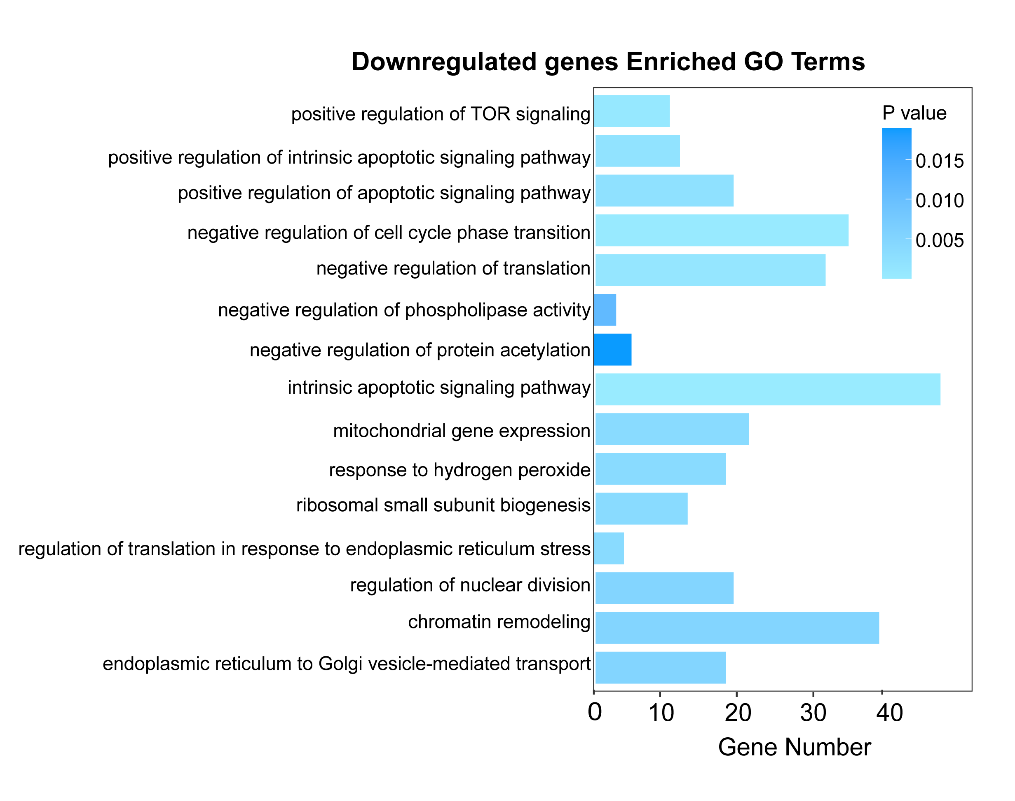


**Figure S24.** Pathways enriched with downregulated differential expression genes of HUVECs between the PBS group and MoS_2_ group using GO analysis.


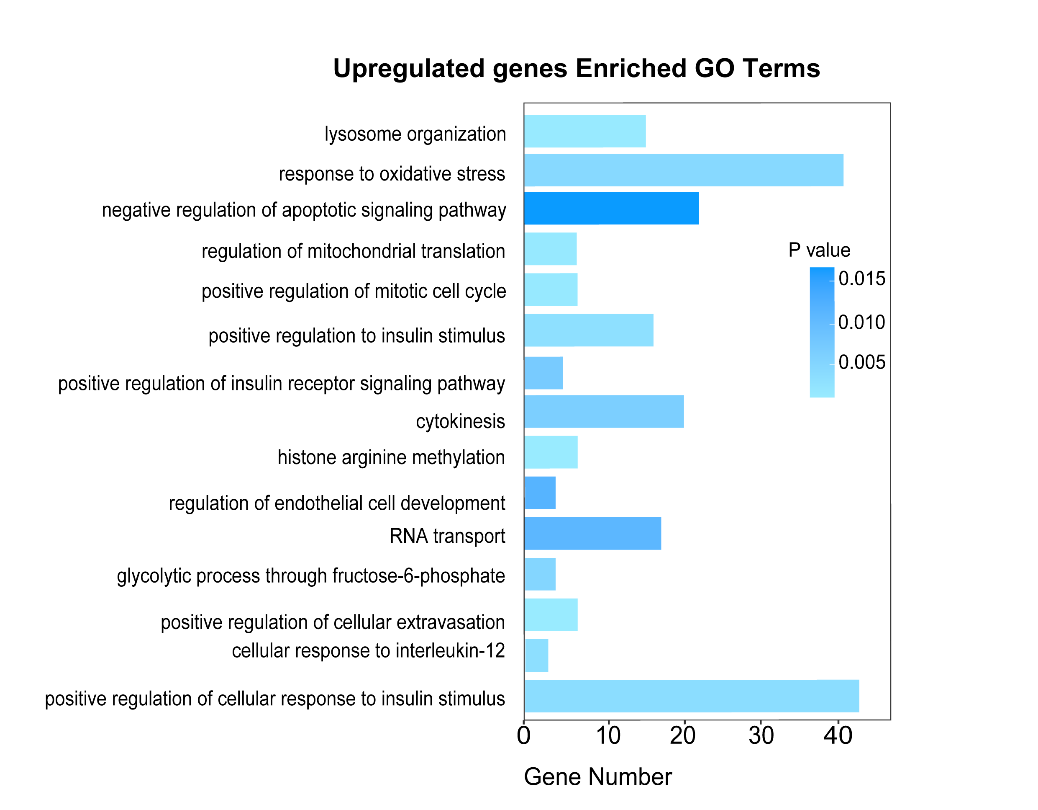


**Figure S25.** Pathways enriched with upregulated differential expression genes of HUVECs between the PBS group and MoS_2_ group using GO analysis.


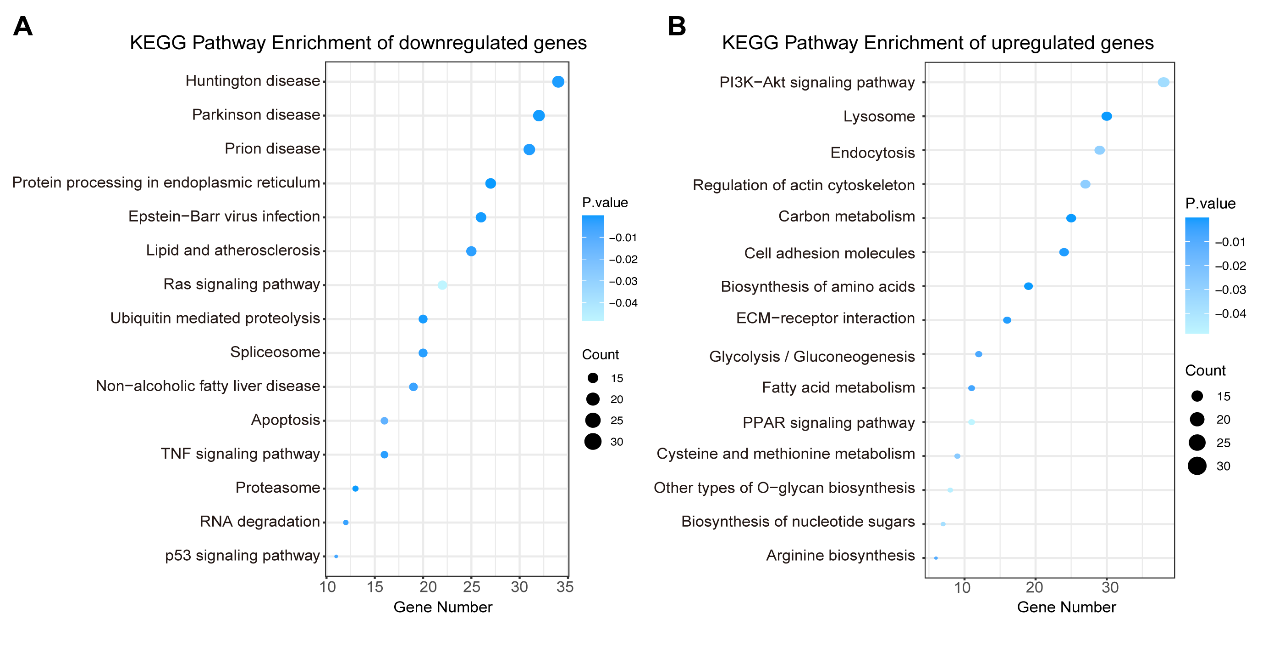


**Figure S26. KEGG enrichment analysis** (A)Pathways enriched with downregulated differential expression genes using KEGG analysis. (B) Pathways enriched with upregulated differential expression genes using KEGG analysis.


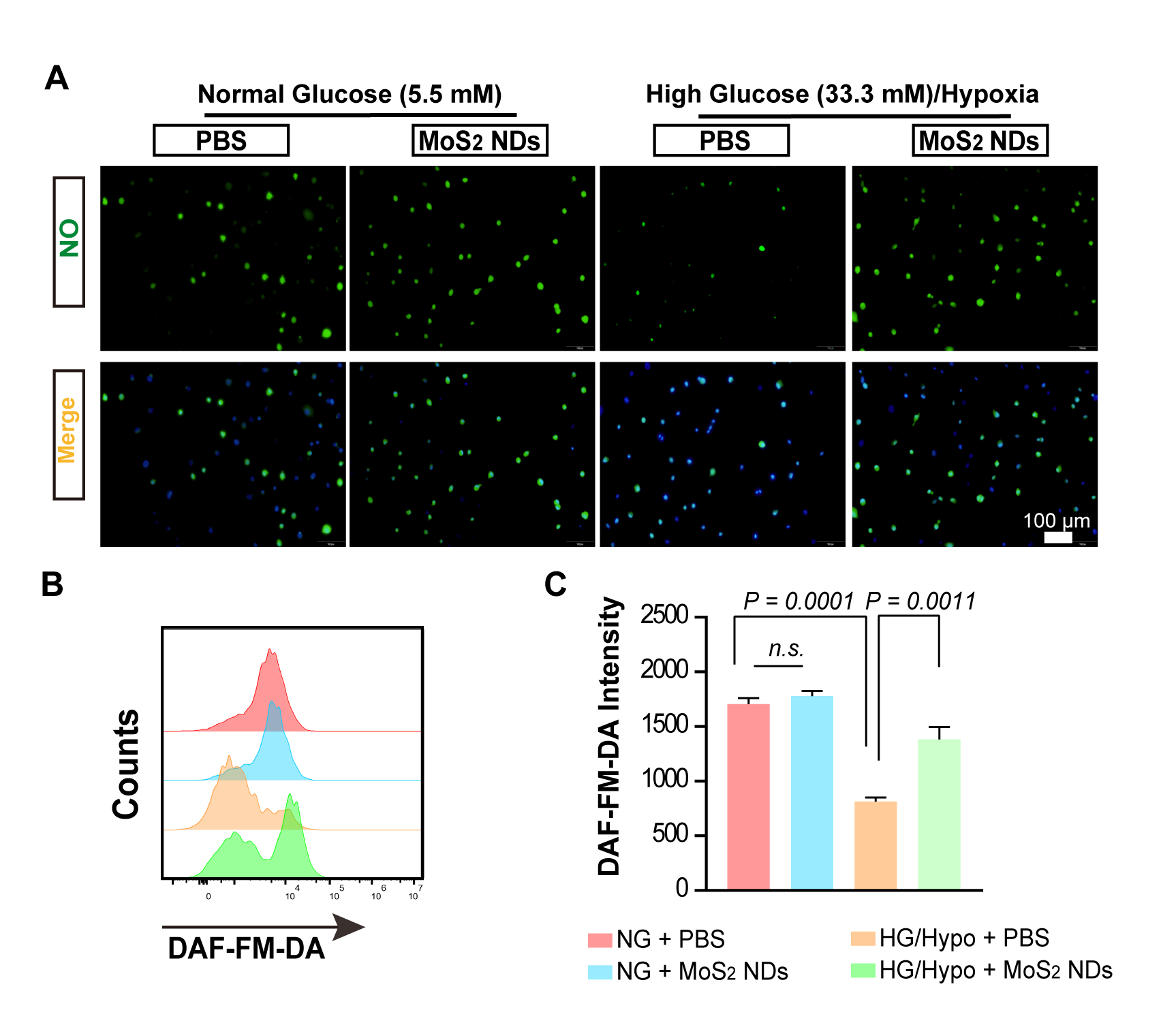


**Figure S27. Intracellular NO level.** (A) Representative images of HUVECs with intracellular NO probe, DAF-FM-DA, with different treatments. (B) DAF-FM-DA fluorescence using flow cytometry. (C) Quantification of the intensity of DAF-FM-DA fluorescence, n = 6, per group. The data are expressed as mean ± SD and P values were calculated by two-way ANOVA with Tukey’s post hoc test.


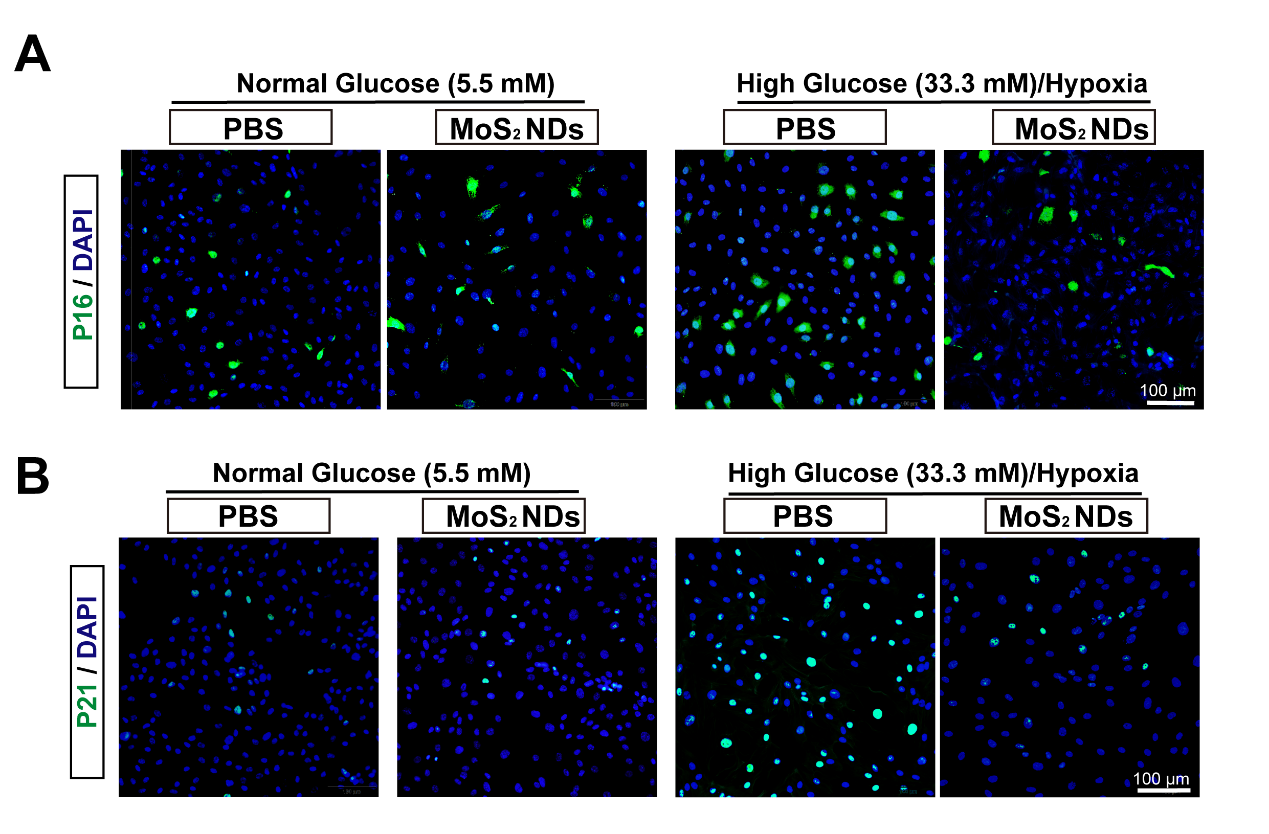


**Figure S28.** (A) p16 and (B) p21 expression shown by immunofluorescence with different treatments.


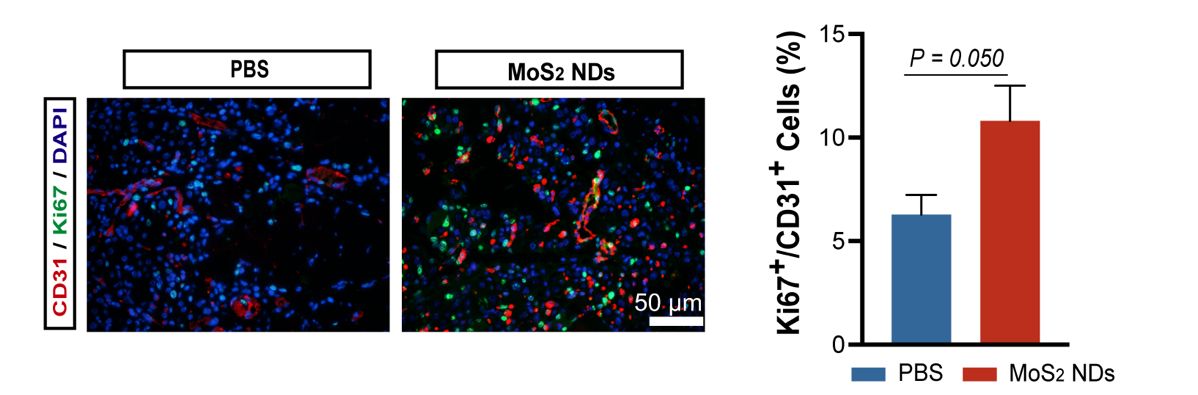


**Figure S29.** Representative CD31/Ki67 immunofluorescent images and quantification of Ki67^+^ /CD31^+^ area of cross sections with PBS and MoS_2_ NDs on day 7. n = 6, per group. The data are presented as the mean ± SD, and P values were calculated by *t*-test.


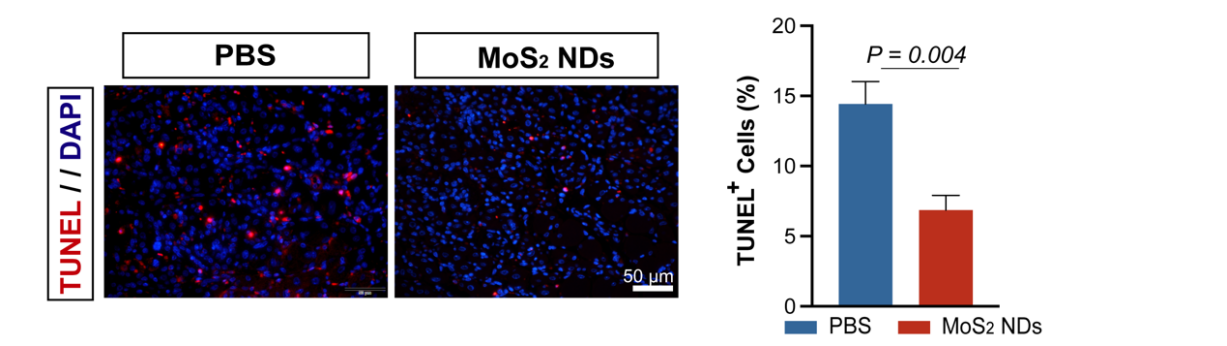


**Figure S30.** Representative TUNEL images and quantification of apoptotic area of cross sections with PBS and MoS_2_ NDs on day 7. n = 6, per group. The data are presented as the mean ± SD, and P values were calculated by *t*-test.


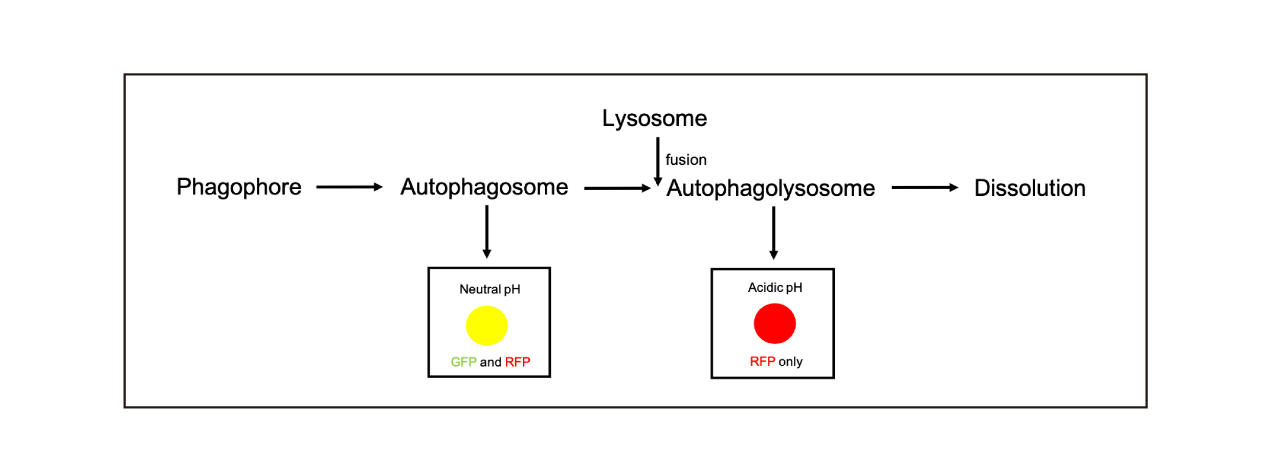


**Figure S31. The rationale of the RFP-GFP-LC3 assay.** Under physiological pH, LC3^+^ vesicles emit both green and red fluorescence. Upon fusion with lysosomes and concomitant acidification, GFP fluorescence is rapidly lost, resulting in vesicles with only RFP fluorescence.


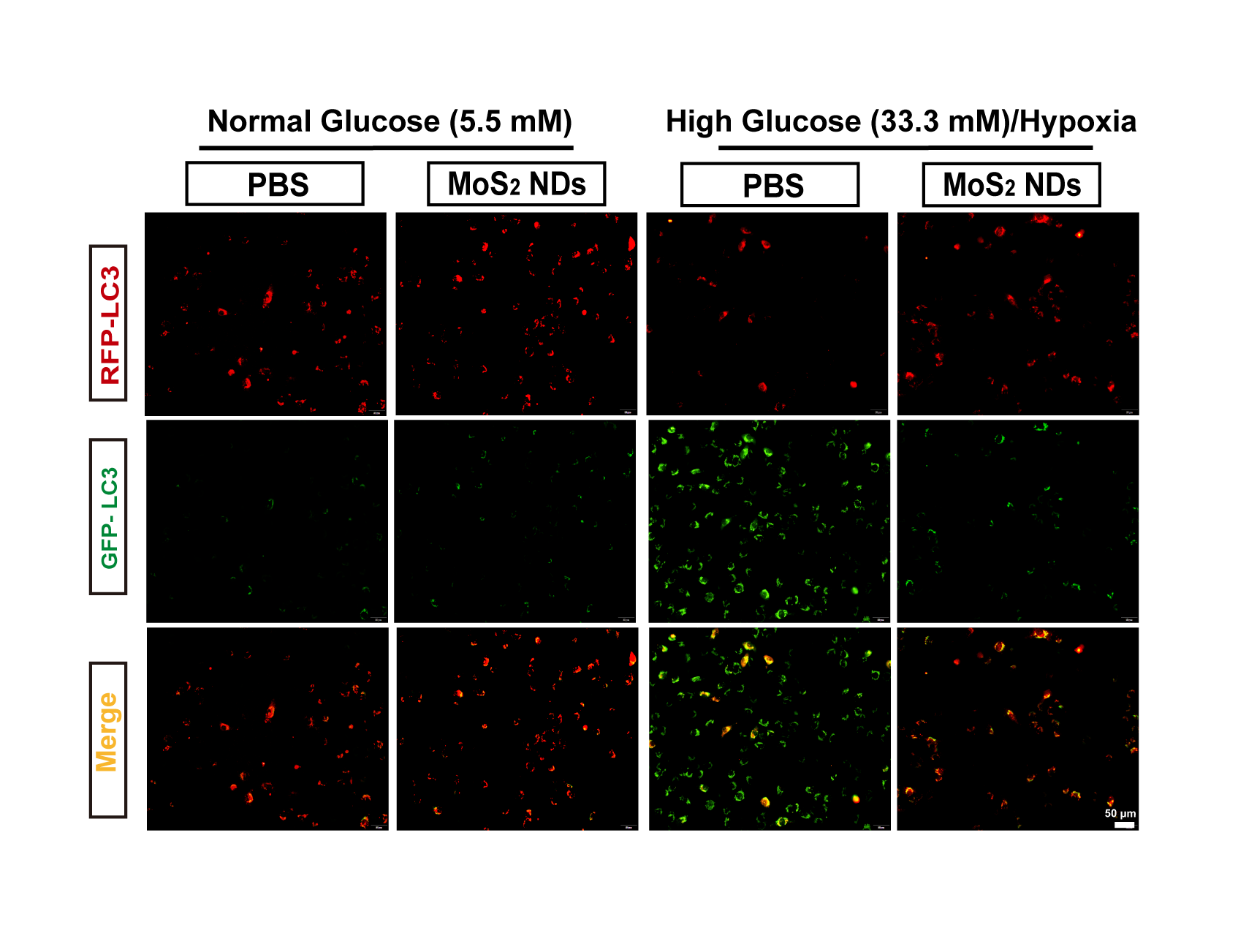


**Figure S32.** Representative images of cells expressing RFP-GFP-LC3 following different treatments by fluorescence microscope.


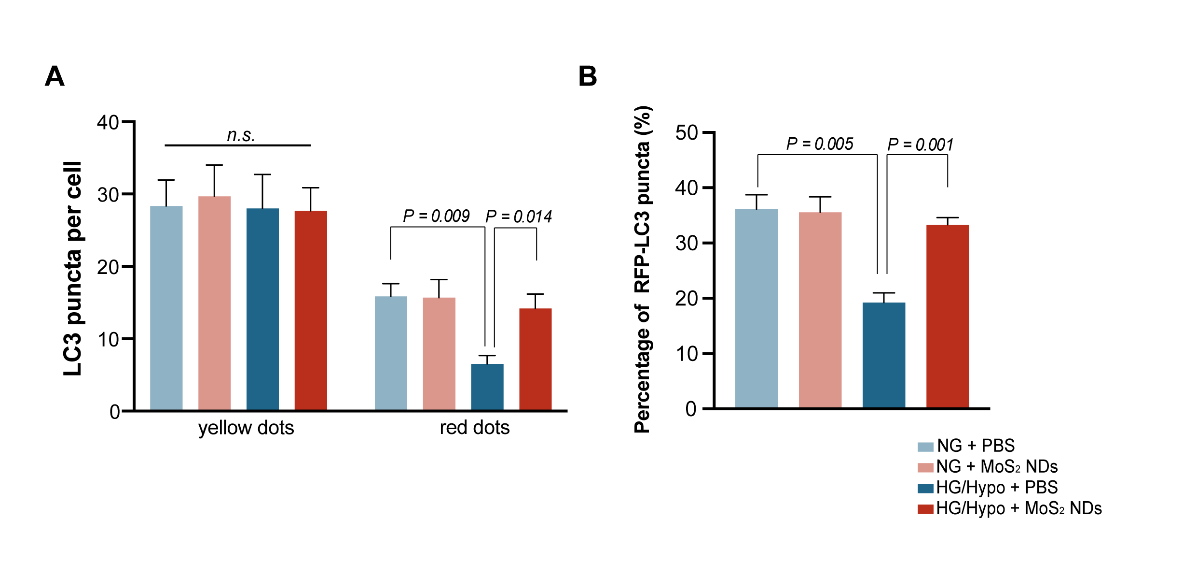


**Figure S33.** (A) Quantification of the number of autophagosomes (yellow dots) and autophagolysosomes (red dots) with different treatments, n = 6 per group. (B) The proportion of autophagolysosomes (yellow dots) in all LC3 puncta, n = 6 per group. The data are presented as the mean ± SD, and P values were calculated by two-way ANOVA with Tukey’s post hoc test.


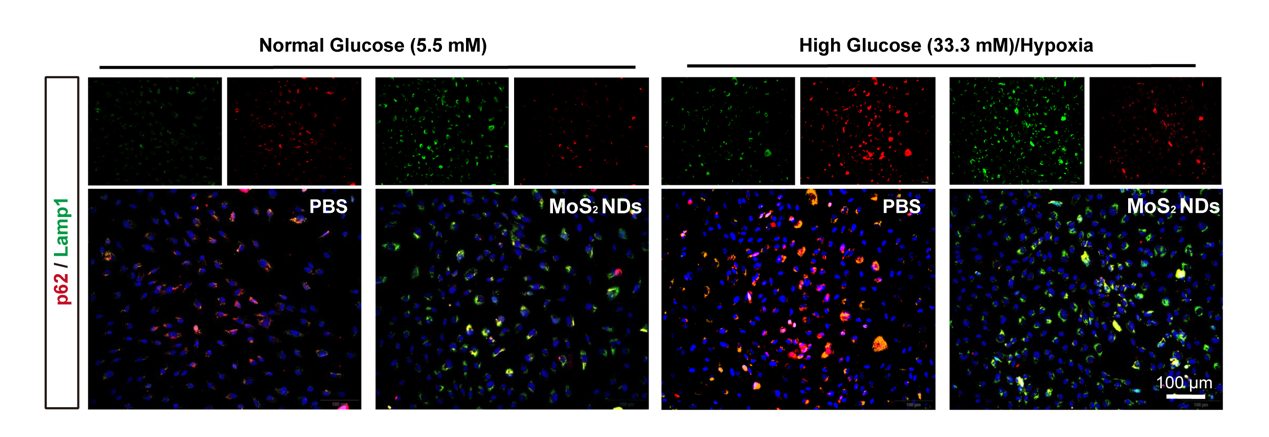


**Figure S34.** Representative images of immunofluorescence staining of p62 and Lamp1 with different treatments.


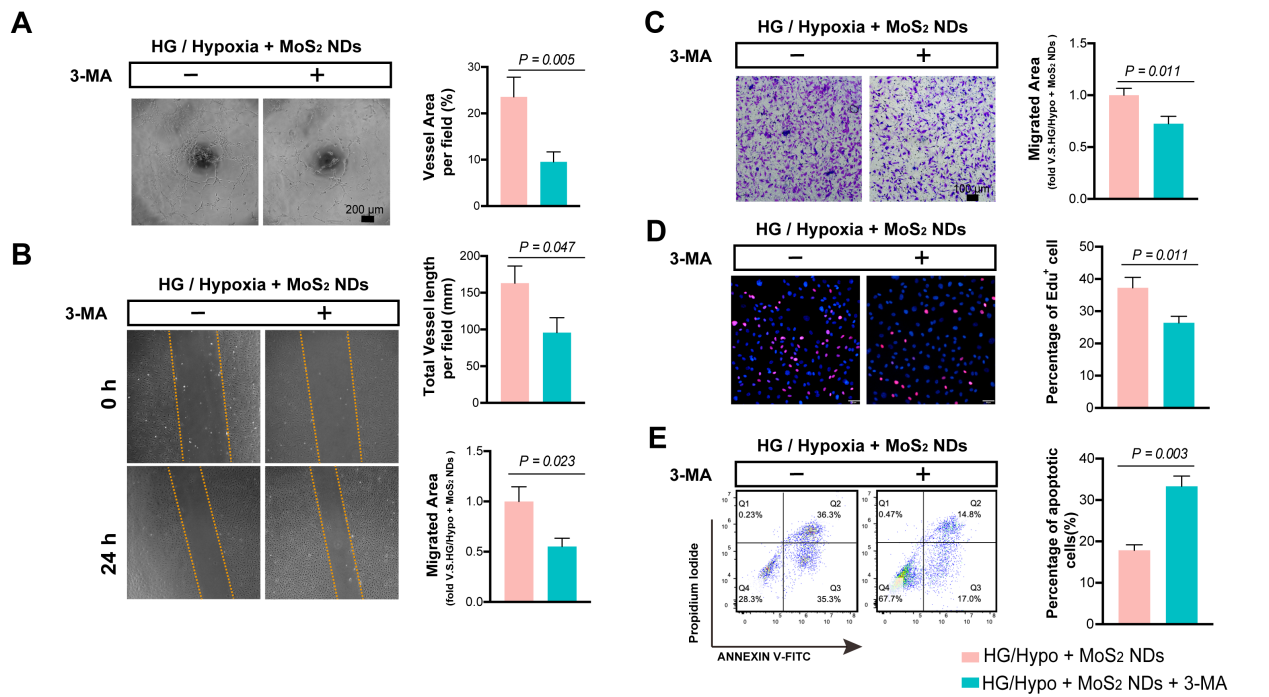


**Figure S35**. **Impaired angiogenesis due to inhibition of MoS_2_ NDs induced autophagy by 3-MA.** (A) Tube formation, (B) wound healing analysis, (C) Transwell analysis, (D) EdU staining, (E) Annexin-V/PI flow cytometric detection of HUVECs under HG/Hypo conditions treated with MoS_2_ NDs with or without the autophagy inhibitor, 3-MA, n = 4, and P values were calculated by *t*-test. All data are expressed as mean ± SD.


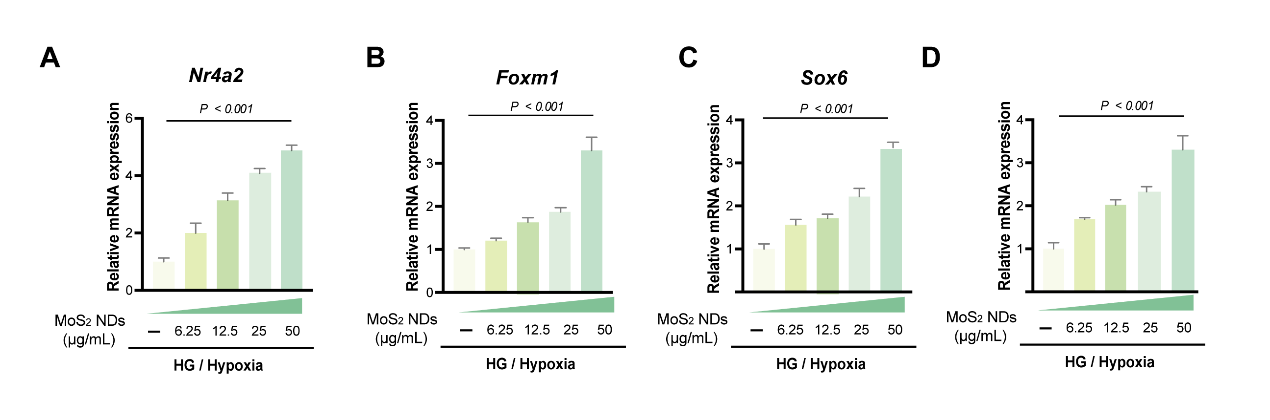


**Figure S36.** Quantification of the mRNA expression levels of TFs with MoS_2_ NDs in a dose-dependent manner, n = 3, per group. The data are expressed as mean ± SD and P values were calculated by one-way ANOVA.


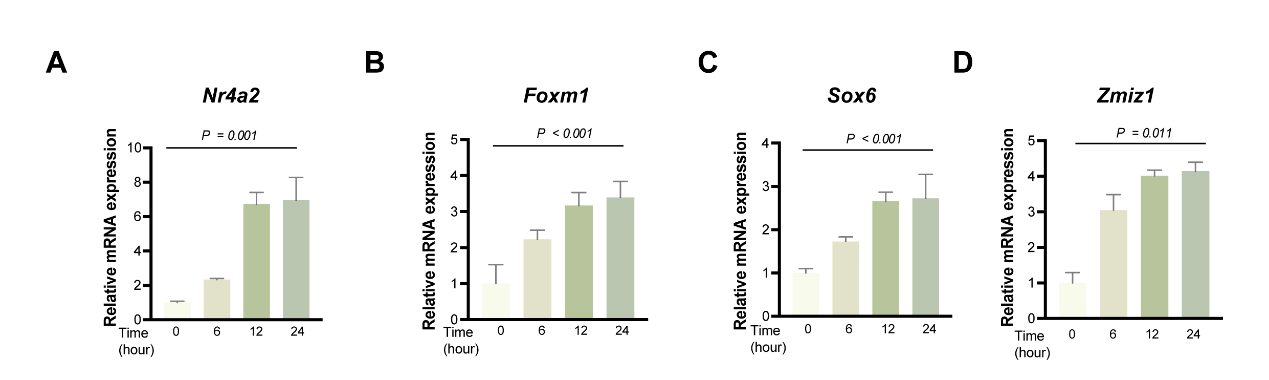


**Figure S37.** Quantification of the mRNA expression levels of TFs with MoS_2_ NDs in a time-dependent manner, n = 3 per group. The data are expressed as mean ± SD and P values were calculated by one-way ANOVA.


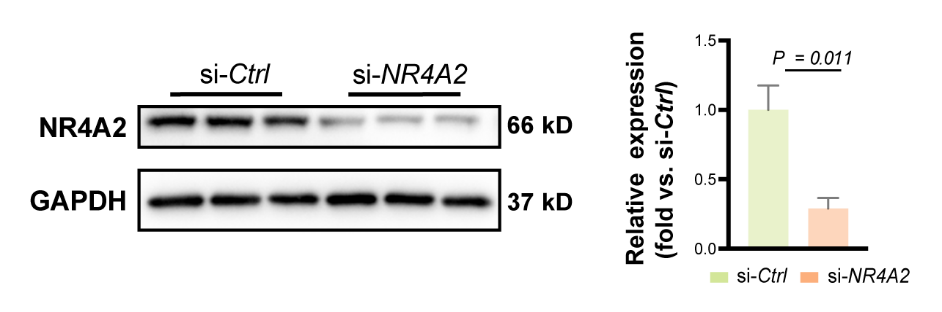


**Figure S38.** The protein expression of NR4A2 with si-*Ctrl* and si-*NR4A2*, n = 3 per group. The data are presented as the mean ± SD, and P values were calculated by *t*-test.


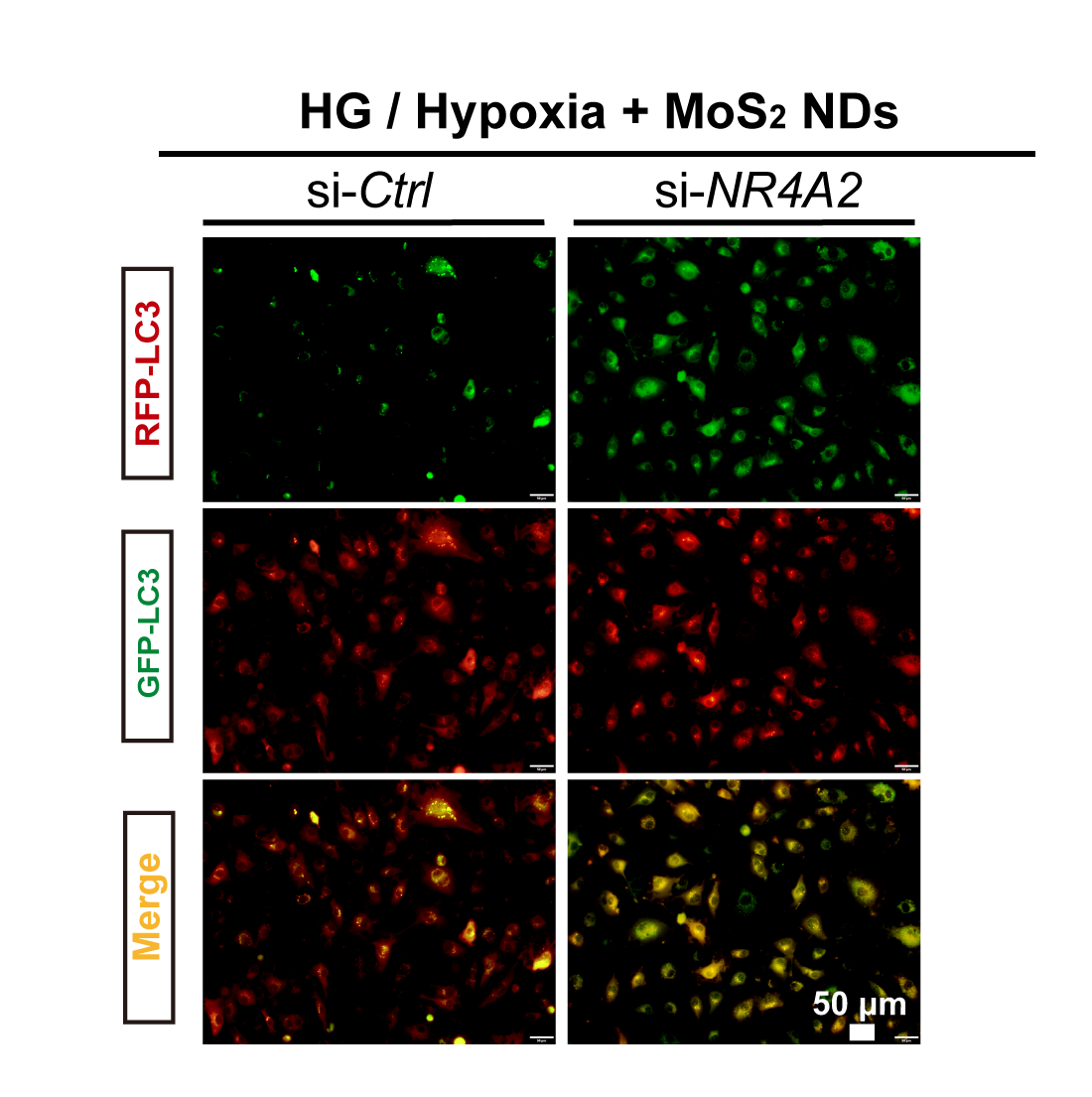


**Figure S39.** Representative images of cells expressing RFP-GFP-LC3 with si-*Ctrl* and si-*NR4A2.*

**Supplementary tables**

**Table S1.** Mo 3d peaks

| PEAK | **Energy (eV)** | chemical state | AC% |
| --- | --- | --- | --- |
| Mo3d5 | 228.58 | Mo-S | 25.71 |
| Mo3d3 | 231.93 |  |  |
| Mo3d5 | 229.98 | Mo(IV)-O | 49.12 |
| Mo3d3 | 233.05 |  |  |
| Mo3d5 | 232.04 | Mo(VI)-O | 25.17 |
| Mo3d3 | 235.09 |  |  |
| S2s | 226.26 | S2s | / |

**Table S2.** Blood glucose of diabetic mice subjected to experiments

|  | PBS group (mM) | |  | MoS_2_ NDs group(mM) | |
| --- | --- | --- | --- | --- | --- |
|  | Two weeks after STZ | Before execution |  | Two weeks after STZ | Before execution |
| 1 | 24.3 | 29.1 | 1 | 21.9 | 17.9 |
| 2 | 25.0 | 26.0 | 2 | 22.6 | 23.4 |
| 3 | 23.1 | 28.2 | 3 | 19.4 | 28.7 |
| 4 | 28.8 | 24.5 | 4 | 19.9 | 21.5 |
| 5 | 31.5 | 22.4 | 5 | 21.4 | 24.9 |
| 6 | 22.0 | 27.3 | 6 | 21.4 | 21.6 |
| 7 | 25.0 | 20.3 | 7 | 22.9 | 22.4 |
| 8 | 18.8 | 16.8 | 8 | 23.1 | 20.7 |
| 9 | 22.6 | 20.1 | 9 | 18.0 | 24.0 |
| 10 | 23.9 | 21.6 | 10 | 21.3 | 24.5 |
| 11 | 22.8 | 19.0 | 11 | 23.3 | 21.9 |
| 12 | 22.0 | 20.0 | 12 | 23.0 | 19.5 |
| 13 | 24.4 | 21,5 | 13 | 28.0 | 19.7 |
| 14 | 19.9 | 24.2 | 14 | 26.9 | 26.6 |
| 15 | 25.7 | 17.1 | 15 | 27.6 | 29.4 |
| 16 | 17.0 | 26.9 | 16 | 32.1 | 27.5 |
| 17 | 20.3 | 29.3 | 17 | 29.2 | 22.6 |
| 18 | 19.0 | 21 | 18 | 21.8 | 20.5 |
| 19 | 24.9 | 22.3 | 19 | 16.4 | 26.1 |
| 20 | 22.5 | 23.1 | 20 | 23.6 | 29.6 |
| 21 | 23.9 | 20.4 | 21 | 16.7 | 16.9 |
| 22 | 22.7 | 22.7 | 22 | 19.2 | 21.7 |
| 23 | 23.3 | 27.1 | 23 | 30.8 | 32.7 |
| 24 | 24.3 | 30.2 | 24 | 35.4 | 22.1 |
| 25 | 29.6 | 26.4 | 25 | 24.2 | 25.7 |
| 26 | 24.2 | 31.7 | 26 | 32.9 | 22.3 |
| 27 | 21.8 | 27.6 | 27 | 27.4 | 27.4 |
| 28 | 26.9 | 24.7 | 28 | 24.3 | 31.6 |
| 29 | 19.8 | 19.2 | 29 | 24.5 | 28.1 |
| 30 | 26.4 | 24.1 | 30 | 25.7 | 25.6 |

**Table S3.** Primers used for RT-qPCR

| Abbreviations | Sequences | |
| --- | --- | --- |
|  | Forward | Reverse |
| *NR4A2*  (*Homo sapiens*) | 5’-TTCCACCAGAACTACGTGGC-3’ | 5’-AAGCGCATCTGGCAACTAGA-3’ |
| *FOXM1*  (*Homo sapiens*) | 5’-AGAAACGACCGAATCCAGAGC-3’ | 5’-GTGGTAGCAGTGGCTTCATCT-3’ |
| *SOX6*  (*Homo sapiens*) | 5’-CTACCTCACCACATAAGCCTGA-3’ | 5’-ACCACATCGGCAAGACTCC-3’ |
| *ZMIZ1*  (*Homo sapiens*) | 5’-CAGCCAACCACAATGACGAG-3’ | 5’-AGACCTCCACATCAGCGTCT-3’ |
| *GAPDH*  (*Homo sapiens*) | 5’-GTCTCCTCTGACTTCAACAGCG-3’ | 5’-ACCACCCTGTTGCTGTAGCCAA-3’ |
